# Transposon expression in the *Drosophila* brain is driven by neighboring genes and diversifies the neural transcriptome

**DOI:** 10.1101/gr.259200.119

**Published:** 2020-11

**Authors:** Christoph D. Treiber, Scott Waddell

**Affiliations:** Centre for Neural Circuits and Behaviour, University of Oxford, Oxford OX1 3SR, United Kingdom

## Abstract

Somatic transposon expression in neural tissue is commonly considered as a measure of mobilization and has therefore been linked to neuropathology and organismal individuality. We combined genome sequencing data with single-cell mRNA sequencing of the same inbred fly strain to map transposon expression in the *Drosophila* midbrain and found that transposon expression patterns are highly stereotyped. Every detected transposon is resident in at least one cellular gene with a matching expression pattern. Bulk RNA sequencing from fly heads of the same strain revealed that coexpression is a physical link in the form of abundant chimeric transposon–gene mRNAs. We identified 264 genes where transposons introduce cryptic splice sites into the nascent transcript and thereby significantly expand the neural transcript repertoire. Some genes exclusively produce chimeric mRNAs with transposon sequence; on average, 11.6% of the mRNAs produced from a given gene are chimeric. Conversely, most transposon-containing transcripts are chimeric, which suggests that somatic expression of these transposons is largely driven by cellular genes. We propose that chimeric mRNAs produced by alternative splicing into polymorphic transposons, rather than transposon mobilization, may contribute to functional differences between individual cells and animals.

Transposons compose up to ∼50% of eukaryotic genomes (Britten and Kohne 1968; Ketchum et al. 2000; International Human Genome Sequencing Consortium 2001), and their mobilization in the germline contributes to chromosome evolution. Transposon activity comprises a wide array of molecular functions (Sienski et al. 2012; Bourque et al. 2018). Nonheritable de novo transposition in neural tissue may contribute to functional heterogeneity in the brain and to neurological disease (Muotri et al. 2005; Coufal et al. 2009; Baillie et al. 2011; Kazazian 2011; Evrony et al. 2012; Kazazian and Moran 2017; Schauer et al. 2018). However, it is difficult to map rare de novo transposon insertions using whole-genome DNA sequencing (Baillie et al. 2011; Evrony et al. 2012, 2016; Perrat et al. 2013; Upton et al. 2015; Treiber and Waddell 2017). Some studies therefore correlate neurodegeneration in animal models with changes in transposon expression (Li et al. 2012, 2013; Krug et al. 2017; Guo et al. 2018; Sun et al. 2018). Using elevated expression as a proxy for mobility could be misleading because it does not always result in de novo somatic transposition (Evrony et al. 2012, 2016; Treiber and Waddell 2017). It is therefore important to understand what controls neural expression of transposon-derived sequences.

Transposons often reside in introns where they can introduce splice sites producing chimeric mRNAs between the transposon and the relevant gene (Makałowski et al. 1994; Deininger 2011). Around 4% of human genes incorporate transposon sequences as novel exons (Nekrutenko and Li 2001), and 75% of human lncRNAs contain segments of transposon origin (Kapusta et al. 2013). However, it is unclear how chimeric transcripts from these loci contribute to the overall pool of transposon mRNAs in somatic cells. Reliable measurement of autonomous and nonautonomous transposon expression in somatic tissue is hampered by repetitive sequences being difficult to map and germline transposons being polymorphic (Lanciano and Cristofari 2020). Hence, many somatic transposon expression studies have analyzed single transposon families or have used bulk sequencing of tissues or cultured cells (Faulkner et al. 2009; Rangwala et al. 2009; Li et al. 2013; Philippe et al. 2016; Wang et al. 2016; Pinson et al. 2018; Babaian et al. 2019; Chung et al. 2019). A genome-wide assessment of the prevalence of chimeric transcripts requires that cellular expression of each transposon in the genome can be related to that of their surrounding genes. Technical developments in high-throughput single-cell transcriptomics of complex tissues, such as the fly brain, make this possible (Macosko et al. 2015; Croset et al. 2018).

Here, we used single-cell RNA-seq (scRNA-seq) to map transposon expression to individual cells in the *Drosophila* midbrain. Combining these data with high-coverage genomic DNA (gDNA) sequencing of the same inbred fly strain permitted neural transposon expression to be correlated with that of genes within which they are inserted. We confirmed these transposon–gene interactions by extracting mRNA from heads of the same strain and performing high-coverage bulk mRNA sequencing. Breakpoint-spanning sequences identified genome-wide splicing of host genes to transposons that generates a considerable diversity of mature chimeric mRNAs. We also present a quality-control approach using immobile genetic elements (IGEs) to quantify rates of amplification artifacts in bulk mRNA sequencing data. Finally, we analyze mRNA sequencing data from other fly strains to assess how chimeric transcripts vary between strains.

## Results

### Single-cell transcriptomics reveals cell type–restricted transposon expression

**Figure 1.**
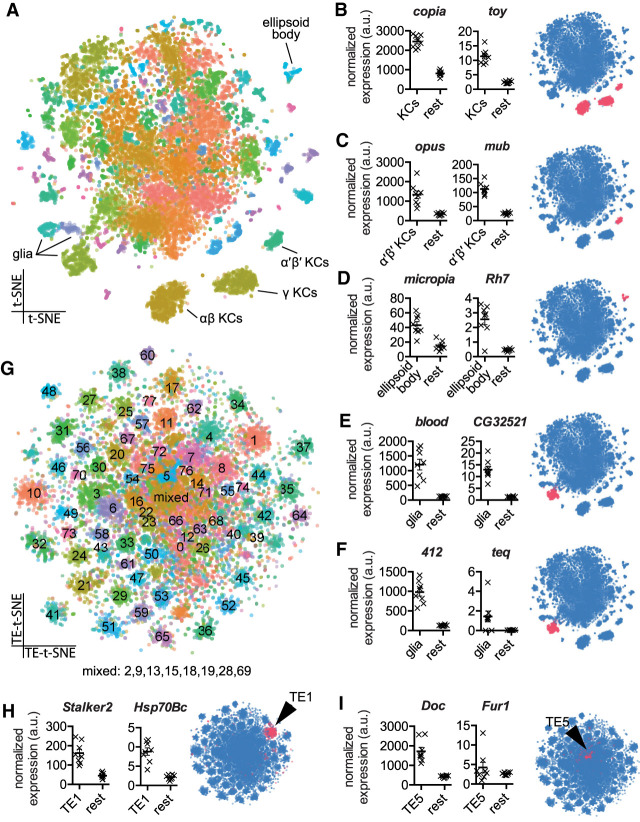
Single-cell transcriptomics reveals patterned transposon expression in the *Drosophila* midbrain. (*A*) Two-dimensional reduction (t-SNE) of 14,804 *Drosophila* midbrain cells based on gene expression levels. Colors represent cell clusters (at SNN resolution of 3.5). (*B*–*F*) Mean expression of transposons and neighboring cellular genes in the relevant cell groups in eight biological replicates and t-SNE representation of cell type–restricted expression. (*B*) *copia* and *twin of eyeless* (*toy*) in all Kenyon Cell (KC) classes. (*C*) *opus* and *mushroom-body expressed* (*mub*) in α′β′ KCs. (*D*) *micropia* and *Rhodopsin 7* (*Rh7*) in the ellipsoid body. (*E*,*F*) *blood* and *CG32521*, and *412* and *Tequila* (*teq*) in glia. Values represent the mean normalized number of unique molecular identifiers (UMI's) in an average cell from each cell type and from the rest of the midbrain. Error bar indicates SEM. Transposon and gene levels were normalized separately. Blue schematic shows location of cell cluster (pink) in t-SNE plot. (*G*) Two-dimensional reduction of 14,804 *Drosophila* midbrain cells based exclusively on transposon expression levels. Colors represent cell clusters (at SNN resolution of 3.5). (*H*,*I*) Mean expression of *Stalker2* and *Heat-shock-protein-70Bc* (*Hsp70Bc*), and *Doc* and *Furin 1* (*Fur1*) in their relevant transposon clusters and the position of the cluster in the overall transposon-based t-SNE (indicated in pink).

The *Drosophila* genome contains at least 112 transposon subfamilies, with copy number of an individual type ranging from a few to hundreds (Kaminker et al. 2002). Conventional scRNA-seq analyses typically discard sequencing reads that align to multiple genomic loci and therefore underestimate transposon expression. Multiply aligned scRNA-seq reads can be kept and their counts divided by the number of copies in the genome. However, germline insertions in the reference genome are likely to differ substantially from insertions in our tested fly strain, making quantification of their expression inaccurate. We therefore devised an alternative analysis pipeline to map expression of all transposons within scRNA-seq data (scTE-seq). scTE-seq masks repetitive sequences in the reference genome and adds a single copy of the consensus sequence for every known transposon to this masked genome. This produces a *Drosophila* reference genome with one copy of each transposon subfamily. We used this modified reference genome to map transposon and gene reads onto individual midbrain cells from a fly strain expressing mCherry in αβ Kenyon cells (KCs) of the mushroom body (MB), henceforth called αβCherry flies. We found evidence for expression of the sense and antisense strands of most transposons, which composed 75.5% and 24.5% (±1.9% SD) of all transposon expression, respectively (Supplemental Fig. S1; Supplemental Table S1). We verified our mapping approach reliably captured transposon reads by comparing our results from scTE-seq to those obtained using RepEnrich2 (Criscione et al. 2014). Counts computed by RepEnrich2 were strongly correlated with the number of uniquely mapping reads identified by scTE-seq (R^2^= 0.661) (Supplemental Fig. S2). Therefore, mapping to consensus sequences did not bias transposon expression levels. We clustered cells from the midbrain and assigned many clusters to known cell types using marker gene expression (Fig. 1A; Croset et al. 2018). Displaying transposons on the cluster plot revealed some to be up-regulated in specific cell types. For example, the long-terminal repeat (LTR) retrotransposons *copia* and *opus* were elevated in the αβ, α′β′, and γ KCs (Fig. 1B, left) and α′β′ KCs (Fig. 1C, left), respectively. Other LTR retrotransposons such as *micropia* were up-regulated in the ellipsoid body (Fig. 1D, first graph), whereas *blood* and *412* were higher in glia (Fig. 1E,F, left).

### Transposon expression correlates with that of cellular genes they are inserted within

We reasoned that transposon expression might be elevated in specific cells because a copy of that transposon is inserted in a gene that is highly expressed in the same cells. To test this hypothesis, we took our previously published high-coverage gDNA sequence of αβCherry flies and mapped the germline transposon insertions in these flies using TEchim, a custom-built transposon analysis program. TEchim uses STAR aligner (Dobin et al. 2013), to screen sequencing data for reads that span the junction between a genomic locus and a consensus transposon sequence, and BLAST (Altschul et al. 1990), to extract information about the transposon insertion site at single-nucleotide resolution. The aim of TEchim is to extract high-fidelity contiguous breakpoint-spanning reads, which distinguishes it from other approaches such as those combined in the integrated analysis pipeline “McClintock” (Nelson et al. 2017). TEchim generates nucleotide contigs from gDNA or cDNA sequencing reads, then creates in silico paired-end reads and screens them for cases in which one end maps to a gene and the mate read maps to a transposon. Because these in silico reads are derived from contiguous sequences, one can refer back to the original reads to determine transposon–gene breakpoint sequence. TEchim also generates sequencing coverage around insertion sites, which permits estimation of the population frequency of germline insertions. Our gDNA data from 10 individual flies revealed a range of population frequencies for transposons in inter- and intragenic regions (Supplemental Table S2). In the subsequent analyses we focus on insertions detected in at least 50% of flies tested. We found highly penetrant *copia*, *opus*, *micropia*, *blood*, and *412* insertions in *twin of eyeless* (*toy*), *mushroom-body expressed* (*mub*), *Rhodopsin 7* (*Rh7*), *CG32521*, and *Tequila* (*teq*), respectively. Expression of these genes mirrored the pattern of the transposon they harbored (Fig. 1B–F, right). Neural expression of these transposons in αβCherry flies therefore appears to be driven by these nearby genes.

We next assessed whether all our annotated transposons showed patterned midbrain expression. Reclustering the scRNA-seq data using transposon expression generated 78 clusters that mostly contained cells from all eight biological replicates (Fig. 1G; Supplemental Fig. S3), indicative of stereotyped transposon expression between different flies from the same strain. Analysis of cellular gene expression across the transposon clusters showed that many clusters preferentially expressed certain genes. For example, the cluster expressing *Stalker2* LTR was enriched for cells also expressing *Heat-shock-protein-70Bc* (*Hsp70Bc*) (Fig. 1H), and cells in the *Doc*-cluster had high *Furin 1* (*Fur1*) (Fig. 1I). Referring back to the gDNA revealed that αβCherry flies harbor a *Stalker2* copy within *Hsp70Bc* and a LINE-like *Doc* element inside *Fur1*. Again, these data suggest expression of *Stalker2* and *Doc* is driven by a neighboring gene.

### Quantitative analysis reveals high-fidelity transposon–gene coexpression

**Figure 2.**
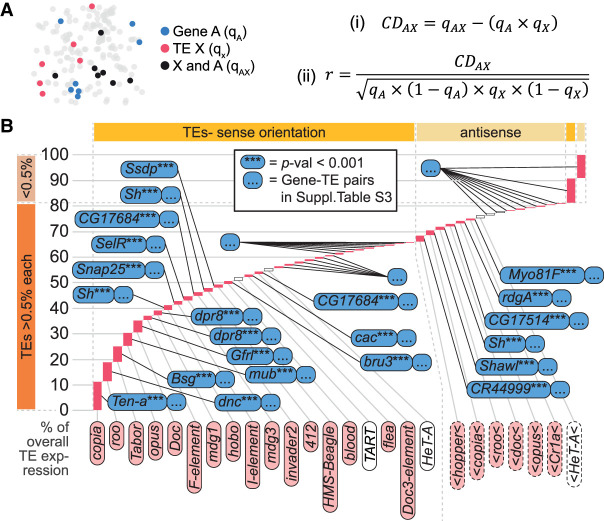
Most transposons are coexpressed with neighboring genes. (*A*) Schematic and formulas describing the calculation of coexpression disequilibrium (*CD*_*AX*_) values. (*B*) Examples of transposon–gene pairs that are neighboring in the genome and coexpressed across the midbrain. Height of pink bars shows relative transposon expression levels in scRNA-seq data. Transposons contributing to >0.5% of overall transposon expression, indicated by dark orange bar on the *bottom left*, are individually displayed, and the associated gene with the lowest corrected *P*-value is indicated for each one. Transposons contributing <0.5%, indicated by light orange bar on the *top left*, are pooled into sense and antisense expression. Transposons are also organized horizontally into sense (*left* side of plot marked with dark yellow bar on *top*) and antisense expressing elements (*right* side of plot, light yellow). See Supplemental Table S3 for the entire list of correlated transposon–gene pairs.

Our gDNA analysis also revealed many transposons inside genes that were more broadly expressed across the brain. In total, we identified 4306 germline transposons (Supplemental Table S2 displays all sites where the upstream breakpoint was detected in at least 50% of flies tested); 2163 of these lie outside and 2143 sit within a gene, henceforth denoted the neighboring gene. Of these, 910 cases were inserted in the same direction as the gene, 1175 in antisense orientation and 58 in loci within genes in both orientations. To quantify the correlated expression of transposons and cellular genes, we devised a method based on the Hardy-Weinberg principle for quantifying linkage equilibrium of two alleles in population genetics (Fig. 2A; Lewontin and Kojima 1960). We binarized our scRNA-seq data to generate the equivalent of biallelic traits in a population (Methods). The proportion of cells expressing a specific transposon was calculated, multiplied by the proportion of cells expressing a certain gene, and then this value was subtracted from the proportion of cells that expressed both the transposon and gene. We termed this value the coexpression disequilibrium (CD). These CD values were normalized to account for variable abundance of each transposon and gene in every transposon–gene pair, and the analysis was repeated for all transposon–transposon and gene–gene pairs. Normalized values were then ranked within each of the eight biological replicates, and *P*-values calculated and corrected for multiple comparisons (Benjamini–Hochberg). These values describe the probability that a transposon–gene pair would have such a highly ranked CD across multiple replicates if they were expressed independently.

We combined the list of all detected germline transposon insertions in αβCherry flies with the scRNA-seq data generated from these flies and calculated CD values between every transposon and its neighboring gene (Supplemental Table S3). For all transposons that contributed to ≥0.5% of overall expression we found at least one copy inside a gene that showed a correlated expression pattern (Benjamini–Hochberg corrected *P* < 0.05) (Fig. 2B). Exceptions were the telomeric *TART*, *TART-A*, and *HeT-A*, which are likely to be autonomously expressed. Transposons inserted in the same orientation as the gene's transcription unit had correlated expression of the sense strand of the transposon with that of the gene. In contrast, the antisense strand was correlated for reverse orientation transposons. Since the number of transposon copies, and therefore the number of potentially correlated neighboring genes, varied between 1 (e.g., *accord*, *1731*, *Tirant*, and so forth) and 91 for *roo* in an antisense orientation, we tested whether the same number of randomly chosen (not neighboring) genes would show similar coexpression patterns with transposons. We randomly selected 10 sets of 2143 genes and counted the number of transposon–gene pairs with correlated expression (below the *P*-value threshold of 0.05) in each gene set. We then performed a χ^2^ test using the mean number of randomly correlated pairs as the expected frequency if there was no interaction between transposons and neighboring genes (Supplemental Table S4). These analyses showed that a neighboring gene significantly influences the expression pattern of almost all transposons in the fly brain.

### Transposons become part of chimeric transcripts with cellular mRNAs

We next tested whether observed coexpression of transposons and neighboring genes might result from chimeric mRNAs formed from the transposon–gene pairs. We extracted mRNA from αβCherry fly heads and generated 250-bp-long reads that were screened for chimeras using TEchim. Incorporating a function in TEchim that maintains strand specificity of input reads enabled unambiguous assignment of chimeras to cellular genes. We found that a large number of intronic transposons give rise to chimeric pre-mRNAs. In total, we retrieved chimeric mRNA segments from 4732 different genomic loci, with 2430 spanning a gene-to-transposon (5′ to 3′) and 2302 a transposon-to-gene junction (Supplemental Table S5). These pre-mRNAs were polyadenylated and frequently contained intron and transposon sequences. Importantly, qPCR-, bulk-, and scRNA-seq analyses would count these transposon-containing pre-mRNAs as evidence for transposon expression. Chimeras included sequences from LTR, LINE-like, and DNA transposons attached to mRNAs from genes involved in many biological processes. For example, we found sequence from the LTR retrotransposon *gypsy* in transcripts of the ubiquitin gene *Ubi-P5E* and of *highwire* (*hiw*), encoding a neuron specific ubiquitin ligase, the non-LTR element *Doc* in *Fur1*, encoding a synaptic membrane bound protease, and the TIR element *hobo* attached to transcripts from *Shaker*, which encodes a voltage-gated potassium channel (Kaplan and Trout 1969; Roebroek et al. 1991; Izquierdo 1994; Wan et al. 2000).

### Immobile genetic elements generate a threshold to exclude amplification artifacts

**Figure 3.**
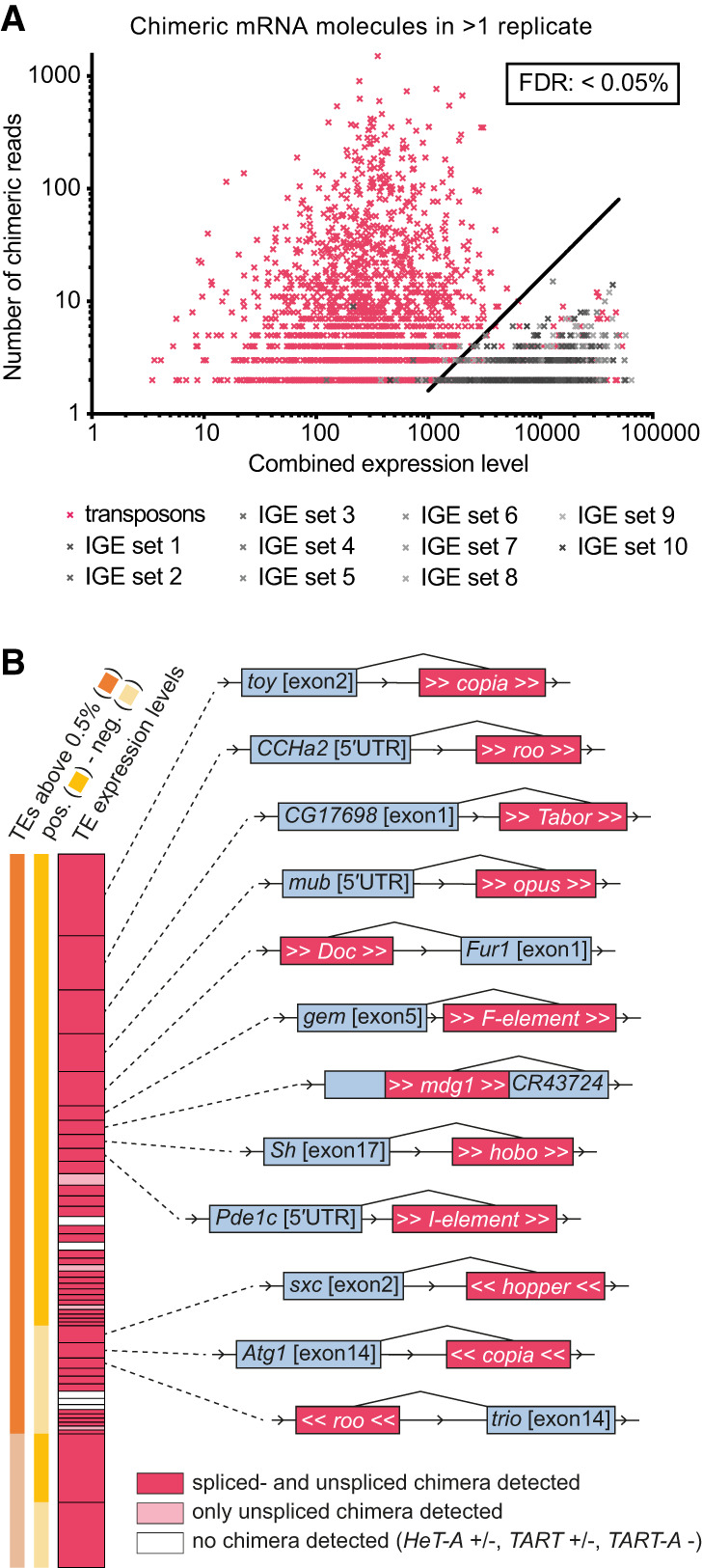
Chimeric transposon–gene mRNA is abundant in the midbrain. (*A*) Graph showing number of chimeric reads, combined expression levels of each transposon–gene pair (pink), and in 10 sets of IGE-gene pairs (gray). Combined expression levels are the square root of the product of reads in our bulk RNA data for both transcripts of a transposon/IGE-gene pair. IGEs were used to calculate a FDR<0.05%. (*B*) Examples of transposon–gene pairs for which chimeric mRNAs were detected. Pink bar represents total transposon expression in scRNA-seq data (as in Fig. 2B), grouped into sense and antisense, as well as contributing to >0.5% and <0.5% of total transposon expression. Dark pink bars indicate that both pre-mRNA and mature spliced mRNA chimeric fragments were detected. Light pink indicates only unspliced chimeras were found. Schematics show splice sites between transposon and the neighboring gene (gray and pink boxes are not to scale). For list of all chimeras, see Supplemental Table S5.

Previous studies of transposon mapping have established that in vitro amplification of DNA often leads to chimeric amplification artifacts (Evrony et al. 2016; Treiber and Waddell 2017). We therefore accounted for similar errors in our mRNA data by calculating the rate of amplification artifacts with 10 sets of 167 exons that were expressed at the same level as each transposon. These exons cannot relocate in gDNA, so we name them immobile genetic elements (IGEs) because IGEs should only occur in one location in gDNA from αβCherry flies. Chimeric reads between IGEs and other genes most likely represent amplification artifacts. As expected, the rate of generating IGE chimeras was correlated to the expression level of the IGE and the gene with which it formed a chimeric molecule. Critically, the IGE chimera rate was substantially lower than that formed between genes and transposons (Fig. 3A). We therefore used prevalence of IGE chimeras to define a false discovery rate (FDR) of 0.05%. The FDR was calculated by dividing the number of IGE chimeras per total chimeras (i.e., including transposon chimeras). This 0.05% threshold resulted in an average of 1.9 IGE hits per 2165 total chimeras (Supplemental Fig. S4; Supplemental Table S6). All chimeric transcripts presented in this study were detected with an FDR <0.05%.

### Many transposons introduce cryptic alternative splice sites into cellular genes

**Figure 4.**
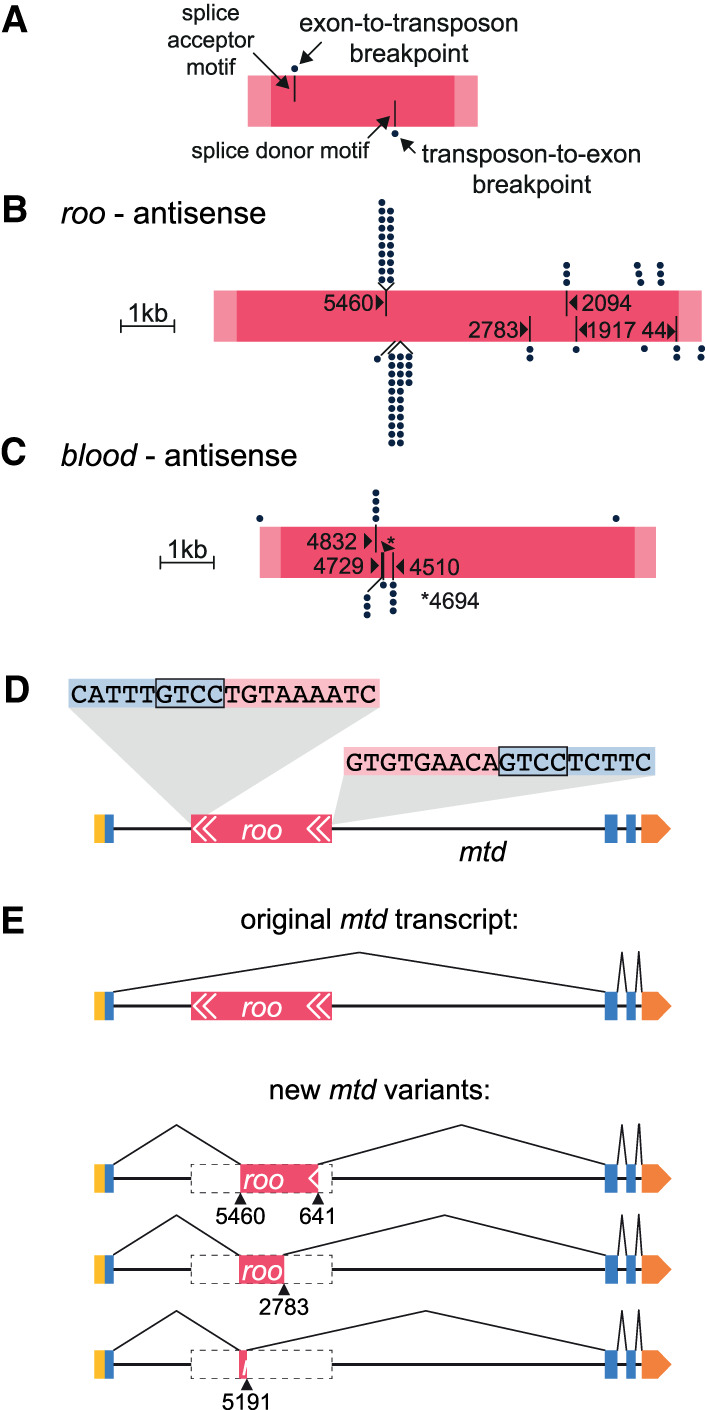
Transposons introduce splice sites at conserved locations. (*A*) Key for labeling scheme in *B* and *C*. Pink bar represents the transposon; light pink ends indicate LTRs, and dark pink indicates the core sequence. Positions of dots *above* the bar represent sites on the transposon where an upstream exon splice donor (SD) has merged. Every dot represents a different gene. Black lines in the *top* half of pink bar represent splice-acceptor (SA) motifs in the transposon. Dots *below* the pink bar indicate location of breakpoints on the transposon that splice to upstream exonic SA sites of different genes. Bars in the *lower* half indicate SD motifs. (*B*,*C*) Representations of antisense *roo* and *blood* (to scale), with all breakpoints to SA and SD sites of neighboring genes. The frequently used site on antisense *roo* at position 5191 is a nonconsensus SD site, lacking the expected GT motif at the immediate breakpoint. The sequence around 5191 resembles a consensus SD motif, although the GT is a GC. Compare TTTGGCAAGTT to motif in Supplemental Figure S5A. (*D*) Illustration of antisense *roo* insertion in the *mustard* (*mtd*) gene. Only one isoform of *mtd* is shown. Yellow box represents 5′ UTR, blue boxes are exons, orange box 3′ UTR, pink represents *roo* transposon with white arrows indicating LTRs. Breakpoint-spanning gDNA reads reveal target site duplication (TSD; *inset*). (*E*) Schematic of original *mtd* transcript and of three new splice isoforms.

Transposon sequences could be removed from the unspliced chimeric pre-mRNAs to yield intact host mRNAs and full-length transposon sequences. However, for most transposon subfamilies, we found at least one neural gene where breakpoint-spanning reads indicate that specific sections of a transposon are spliced into host-gene transcripts (Fig. 3B; Supplemental Table S5, spliced insertions are labeled in column 2). Analysis of the breakpoints inside transposons at these 264 sites revealed that chimeras were formed at conserved locations in each transposon type. For example, where antisense *roo* resided within an intron, we found transcripts where the 3′ end of an upstream exon was fused to either a section of *roo* beginning at position 5460 (for 19 different loci) or 2094 (three loci), and also at several additional breakpoints with lower frequency (Fig. 4A,B). In addition, we identified transcripts where sections of *roo* were bound to the 5′ end of a downstream exon. 3′ Breakpoints at position 5191 of *roo* spliced into transcripts of 24 genes, two genes from position 2783 of *roo*, and several others from unique positions in *roo*. (Note numbering runs backward because it relates to forward orientation of *roo*.) Whereas intronic antisense *roo* provided gene-to-transposon breakpoints for 28 exons, and transposon-to-gene breakpoints for 33, intronic sense *roo* only introduced 4 and 1, respectively (Supplemental Table S5). Similarly, the LTR *blood* contributed more breakpoints when inserted in antisense orientation relative to the host gene (14 vs. six) (Fig. 4C; Supplemental Table S5).

We screened transposon sections around breakpoints for consensus splice-acceptor (SA) and donor (SD) sequence motifs. Often gene-to-transposon chimeras formed at SA consensus motifs, and transposon-to-gene chimeras at SD motifs (Supplemental Fig. S5; Supplemental Table S7; Stephens and Schneider 1992). For example, all breakpoints in antisense *blood* formed with more than one exon were precisely located at predicted SA and SD splice sites (see vertical lines in Fig. 4C). A consensus SD motif was not evident at position 5191 of antisense *roo*, although it frequently provided 5′ sequence to transposon-to-gene chimeric RNAs (Fig. 4B). However, sequence around position 5191 resembles the consensus, with exception of a GT-to-GC conversion (Supplemental Fig. S5). Taken together, our analysis revealed that transposons introduce many alternative splice sites, which are recognized by the host cell spliceosome to join cellular exons to sections of transposon.

We also identified alternative splicing to different sites within the same transposon insertion. Again using *roo* as an example, αβCherry flies harbor an intronic reverse orientation *roo* in the pan-neurally expressed *mustard* (*mtd*) gene, which to date has only been implicated in innate immunity (Fig. 4D; Wang et al. 2012). The wild-type *mtd* locus produces many splice variants, and RNA-seq revealed a complex collection of additional *mtd* splice variants that incorporated different *roo* fragments (Fig. 4E). SD sites upstream of this *roo* came from either *mtd* exon 11 or 13 (annotated exons are numbered backward), and these spliced to the SA at position 5462 within *roo* (Fig. 4E). Three different SD sites (at positions 641, 2784, and 5191) within *roo* spliced out to the closest downstream SA (exon 6) of *mtd*. This *roo* substantially increases the *mtd* mRNA isoform repertoire; without *roo* the locus can express 23 *mtd* isoforms, but with *roo* it can generate 68 differentially spliced mRNAs.

The transcript diversity of 263 other genes was similarly increased by a transposon. These transcripts incorporate 66 different transposon families with each introducing cryptic SA and/or SD sites into host genes (see Supplemental Table S5). For example, chimeric reads indicate that transcription of *Dscam2*, which encodes the transmembrane Down Syndrome cell adhesion molecule 2, is frequently initiated inside a sense insertion of *blood* that spliced into exon 33 (the second exon) of *Dscam2*. This splicing combines ORF2 of *blood* with the remaining *Dscam2* exons and aligns the reading frames, generating a novel N terminus (Supplemental Fig. S6). We also found evidence of transposons resulting in exon skipping (e.g., *412* inside *Tequila*) (Supplemental Fig. S7). Most transposon chimeras resulted from intronic insertions. However, an exonic *hobo* in the *CG31705* gene introduced a cryptic SA spliced to the upstream SD from the first *CG31705* exon, creating a truncated mRNA (Supplemental Fig. S8). These data show that many *Drosophila* transposons are alternatively spliced into cellular mRNAs increasing the isoforms of a large number of neurally expressed genes.

### Alternative splicing into and out of transposons can be highly penetrant

**Figure 5.**
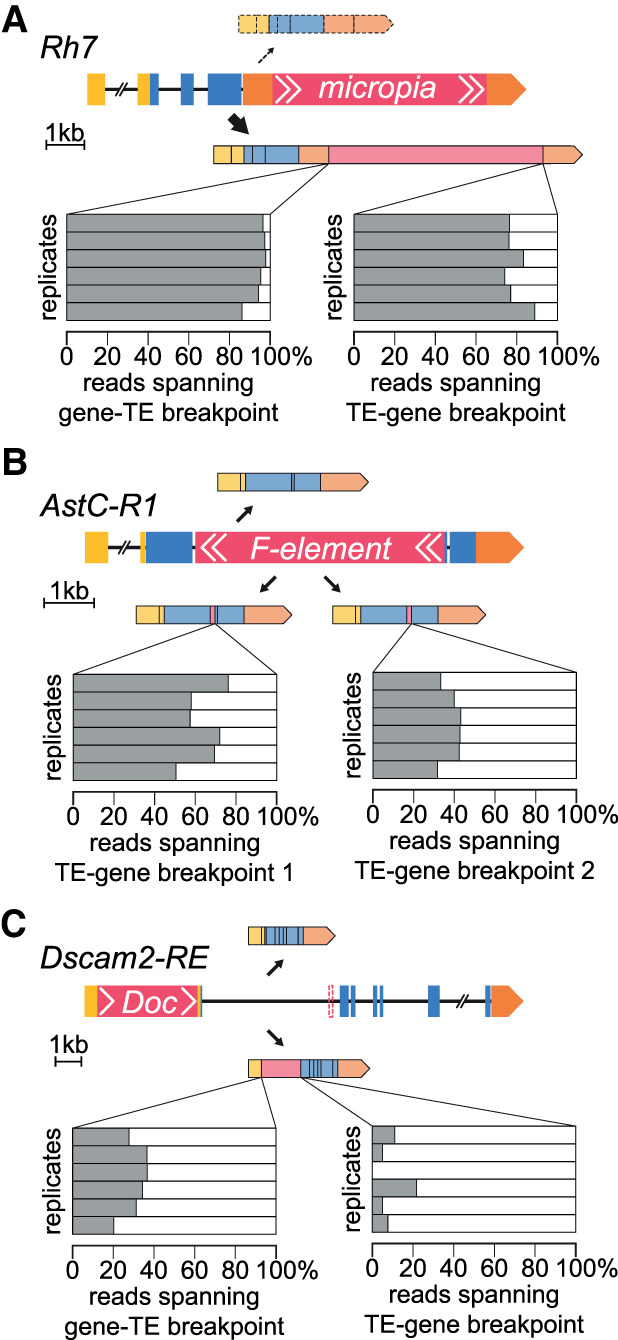
High penetrance of transposon-containing splice isoforms. (*A*) Schematic showing *Rhodopsin 7* locus harboring a sense *micropia* in the 3′ UTR and two splice isoforms. Gray bars show percentage of reads spanning the gene-TE (*left*) and TE-gene (*right*) breakpoint in each of the six tested replicates. (*B*) The *AstC-R1* gene harbors an antisense *F-element* immediately upstream of the second exon that introduces cryptic splice sites. Three spliced isoforms are shown. (*C*) *Dscam2* harbors a sense *Doc* in its 5′ UTR (in addition to a *blood* insertion in its first intron) (Supplemental Fig. S6). For ease of visualization, only the shortest *Dscam2* isoform, RE, is depicted. The *blood* insertion is indicated with a dashed box. See Supplemental Table S5 for complete list.

Chimeric transcripts could be inconsequential if they only constitute a small percentage of the overall transcript repertoire of a gene. To quantify how frequently a transposon-harboring gene produces chimeric mRNAs, we analyzed loci where a transposon splices into an exon–intron junction. For each gene we counted the number of reads spanning the transposon-exon boundary, and the number spanning the exon immediately upstream of and downstream from the transposon. For some genes, most mRNAs contained transposon sequences. For example, 95.3% of all *Rhodopsin 7* transcripts included *micropia* in the 3′ UTR (Fig. 5A), and all mRNAs of the *Allatostatin C receptor 1* (*AstC-R1*) contained a section of *F-element*, spliced into one of two different SA sites in the gene (Fig. 5B). In addition to the *blood* insertion in *Dscam2* mentioned above, we also found a *Doc* insertion in *Dscam2*, which contributed to around a third of all transcripts initiated at the *Dscam2* transcription start site (Fig. 5C). We also found a sense-orientation *flea* in the X-linked *cacophony* (*cac*), which encodes a voltage-gated calcium channel (Smith et al. 1996). This *flea* insertion truncated 12.4% of *cac* transcripts, potentially deleting the last 8–11 coding exons and suggesting that many αβCherry males are likely mutant for the *cac* gene (Supplemental Fig. S9). Another interesting example on the X Chromosome of αβCherry flies is a sense *opus* insertion in *Beadex* (*Bx*), which encodes a long-term memory relevant LIM-type transcription factor (Hirano et al. 2016). This *opus* produces at least two new *Bx* mRNAs (Supplemental Fig. S10), which constitute 4.9% of all *Bx* transcripts. On average, transposons contributed 11.6% of transcripts derived from a gene (Supplemental Table S5).

### Splicing into transposons is common and varies between strains

Transposons are highly variable between fly strains. We therefore analyzed three previously published mRNA sequencing data sets from other fly strains for chimeric transposon–gene mRNAs (MacKay et al. 2012; Croset et al. 2018; Hemphill et al. 2018). Although these prior studies generated shorter paired-end RNA-seq reads, we still found chimeric mRNAs in all three data sets (Supplemental Table S8). Some chimeras were conserved across all strains, whereas others appeared to be strain specific. Of the 1332 chimeras identified in at least two samples of αβCherry flies, 466 were present in at least one of the three other strains and 92 occurred in all four strains. Chimeras that were not detected in other strains could indicate genomic heterogeneity between strains or absence of evidence resulting from lower sequencing coverage. Nevertheless, these results show the prevalence of cellular mRNAs containing transposon sequence.

### Transposon expression is predominantly nonautonomous

**Figure 6.**
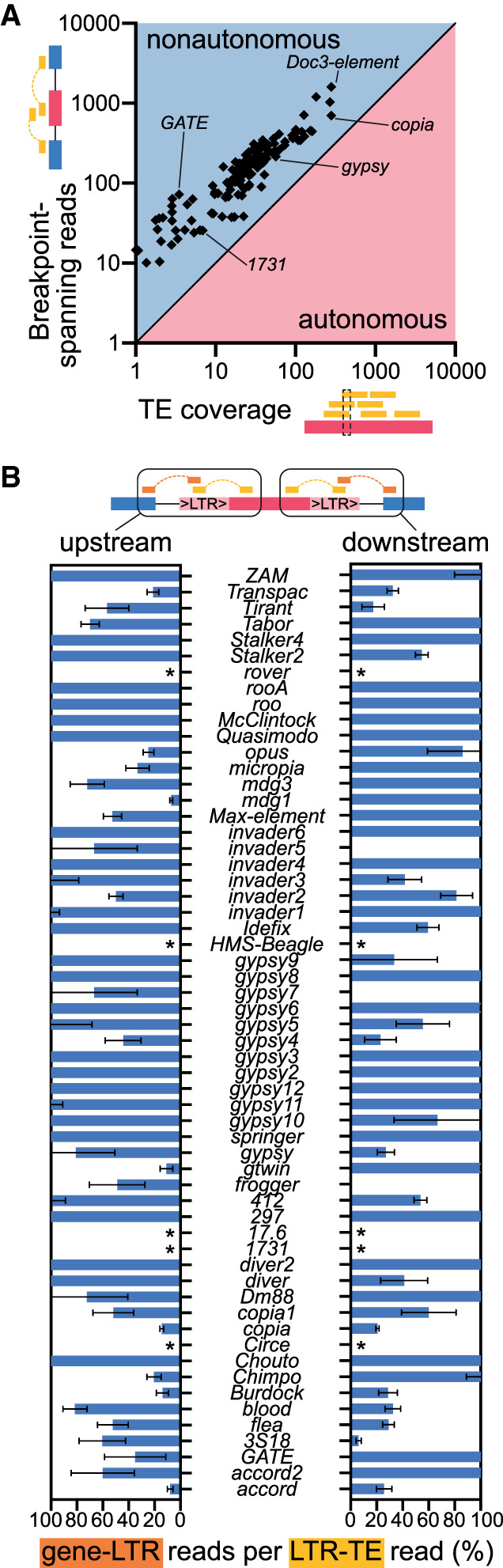
LTR retrotransposon expression is predominantly nonautonomous. (*A*) Plot showing the average number of reads per nucleotide (*x*-axis) and total number of transposon–gene spanning reads (*y*-axis) for every tested transposon. Number of spanning reads is higher for every transposon. (*B*) List of all LTR retrotransposons analyzed in mRNA data. LTR-gene spanning reads were identified for every LTR transposon expressed in the midbrain. Numbers represent percentage of reads spanning LTR-gene versus LTR-TE breakpoints. Values are capped at 100%, but some transposons produced more LTR-gene than LTR-TE reads (Supplemental Table S10). Error bars represent standard deviation. (*) Transposon reference sequences did not contain LTR sections for the five transposons.

Finding that neural expression of consensus transposon sequences is highly correlated with at least one neighboring gene, and that most transposon sequence is part of spliced chimeric mRNAs, implies that expression is largely driven by neighboring genes. Testing this hypothesis further requires comparing the number of reads mapping to a specific transposon with the abundance of breakpoint-spanning reads for that same transposon. However, most transposons are multicopy (six is the median copy number in αβCherry flies) (Supplemental Table S3), so a read mapping inside a transposon cannot be assigned to a specific copy. To overcome these challenges, we quantified the average number of reads that only map to a given transposon consensus sequence (TE-only) per nucleotide for each transposon across our six biological replicates. Next, we counted the number of locus-specific reads that span each transposon and a genomic region (TE-gene). We reasoned that autonomous transposon expression should exclusively generate TE-only reads, whereas nonautonomous expression driven by a neighboring gene should generate similar numbers of TE-only and TE-gene reads. The number of TE-gene reads was higher than the average number of TE-only reads (normalized to transposon length) for every transposon tested (Fig. 6A; Supplemental Table S9), suggesting expression is nonautonomous. We also tested autonomous versus nonautonomous expression by analyzing transposons with LTRs at both ends. Autonomous expression of LTR elements should not result in reads upstream of the element's 5′ LTR (5′-gene-LTR-3′ reads). Quantifying the ratio of 5′-gene-LTR-3′ reads and 5′-LTR-TE-3′ reads revealed that most LTR retrotransposons expressed in the head generate roughly equivalent numbers of each fragment. Breakpoint-spanning reads at the 3′ ends of LTR retrotransposons revealed a similar situation (Fig. 6B; Supplemental Table S10). These analyses provide further evidence that LTR transposons are predominantly expressed as chimeric mRNAs with cellular genes, rather than as autonomous elements.

## Discussion

Combining single-cell expression data from the *Drosophila* midbrain with high-coverage gDNA sequence of the same fly strain revealed that most transposons are expressed as parts of chimeric mRNAs with cellular genes.

Several prior studies have documented that transposons are transcriptionally active in somatic tissue. These reports used methods that either generate cDNA fragments (RNA-seq) (e.g., De Cecco et al. 2013) or amplify short sections of transposon mRNAs (RT-qPCR) (e.g., Li et al. 2013; Guo et al. 2018; Sun et al. 2018). However, these approaches cannot distinguish between autonomous transposon expression and chimeric transposon–gene mRNAs investigated in this study. Baseline and changing cell-specific expression of host genes that produce chimeric transcripts with transposons could therefore be misinterpreted as cell-restricted autonomous transposon expression with potential for mobilization.

Some studies of transposon expression use cap analysis gene expression (CAGE) to distinguish pre-mRNA from 5′ ends of mature mRNAs (Faulkner et al. 2009). Although CAGE reads mapped to transposons represent transcripts where transcription started within a transposon, we identified 243 chimeras that initiated inside (or at the start of) a transposon and spliced into a downstream exon of a gene (Supplemental Table S5). Short 5′ end CAGE reads would rarely identify such chimeric transcripts. In theory, a combination of long-read sequencing—for example, Pacific Biosciences (PacBio) (Rhoads and Au 2015) or nanopore (Deamer et al. 2016)—and ways to identify 5′ caps and 3′ poly(A) tails could discover full-length transposon mRNAs.

Our study illustrates the utility of the *Drosophila* brain to study genome-wide expression of transposons. The single-cell atlases of the entire brain allow transposon expression to be assigned to specific cell types (Croset et al. 2018; Davie et al. 2018; Konstantinides et al. 2018; Allen et al. 2020). This is made easier by transposon subfamilies in *Drosophila* being very discrete, with even related elements having different sequence. In addition, some of these transposons are low copy and even detected within one gene (Supplemental Table S3). This makes it simple to map their expression to cells and to significantly correlate their expression to that of a neighboring gene. In contrast, for a high copy number transposon resident in more than 10,000 genes (cf. LINE-1 in most mammalian genomes), it becomes impossible to distinguish a correlation from chance, because the transposon expression would also be correlated with at least one of 10,000 randomly chosen genes.

We complemented scRNA-seq analyses of transposon coexpression with neighboring genes with discovery of more than 833 chimeric transposon–gene transcripts using bulk RNA-seq. Chimeric transposon–gene fragments were identified in previous studies, with some focusing on individual genes and others analyzing exonized transposons genome-wide (Nekrutenko and Li 2001; Van De Lagemaat et al. 2003; Kapusta et al. 2013). However, to our knowledge, no other study investigated the proportion of transposon expression in somatic cells that comprises exonized transposon fragments. We found that transposon exonization is highly prevalent in the *Drosophila* brain and is likely the main driver of somatic transposon expression. Because we mapped reads to consensus transposon sequences, we may have missed exonization of older transposons that have accumulated many mutations.

We introduce three new pieces of software that should be helpful to other researchers in the field. Although they were developed to analyze *Drosophila* data, they can be readily adapted for sequence data from other species. The three main components are (1) scTE-seq, a tool to map scRNA-seq data onto a masked reference genome and consensus transposon sequences, (2) scRNA-seq-Hardy-Weinberg (scHW), which implements the new method presented here to analyze expression correlations, and (3) TEchim, which combines all analysis steps for identification, characterization, and quantification of chimeric transcripts in bulk mRNA sequencing data and includes IGE analysis to determine the rate of amplification artifacts for each sample.

We found the expression of many transposons to be restricted to small groups of cells. For example, *blood* was highly expressed in most glia, but silent in neurons. In contrast, *gypsy* was detected in some neurons but was absent in glia. Somatic transposition in neurons and glia has been implicated in age-dependent neuronal decline in wild-type and disease models of *Drosophila* (Li et al. 2013; Guo et al. 2018; Sun et al. 2018). Our results constrain these models because mobilization can only occur in cells that express full-length elements or transposon mRNAs that encode enzymes permitting other elements to move in *trans*. Therefore, the *gypsy* retrotransposon is only likely to mobilize in glia if the fly strain studied harbors a copy of *gypsy* in a glial-expressed gene (Krug et al. 2017). Expression below that typically detectable using scRNA-seq could generate full-length transposon mRNAs that reintegrate in the genome. For example, two LINE-1 elements on human Chromosomes 8 and 13 were shown to mobilize in the human brain (Evrony et al. 2015; Sanchez-Luque et al. 2019). However, data in this study, which include higher coverage bulk sequencing data, and our earlier study of the rate of somatic transposition (Treiber and Waddell 2017) indicate that transposon transcripts in the fly brain most frequently represent diversification of the neural transcriptome rather than mobilization.

At this stage we are unable to conclusively show the biological impact of transposon–gene chimeras. The process of transposable elements acquiring new cellular functions that benefit the host cell has been coined transposon “exaptation” (Gould and Vrba 1982). A striking example of this is the neuronally expressed *Drosophila* and rodent Arc proteins, which resemble Ty3/*gypsy* retrotransposon-encoded *gag*. Arc also forms virus-like capsids and binds sequences in the 3′ UTR of *Arc* mRNAs, which enables their intercellular transport (Zhang et al. 2015; Ashley et al. 2018; Pastuzyn et al. 2018). We found a broad range of neural genes for which a substantial proportion of their mature mRNA transcript pool contained transposon sequences. Sometimes transposon sequence is within the open reading frame, and other times it is positioned in 5′ or 3′ UTRs where it could alter traffic and/or translation. However, it is difficult to determine the whole-genome functional consequence of splicing into transposons because we often only retrieve the sequence across the splice junctions. Furthermore, although each transposon has a known consensus sequence, individual copies are polymorphic. Nevertheless, our sequencing shows that transposon exonization often truncates and/or changes the amino acid sequence of the encoded proteins, potentially changing structure and function. We also identified several examples in which inclusion of transposon sequence conserved the reading frame of the host gene and may generate a novel chimeric protein. Among the 264 transposon-harboring genes identified in this study, there are several that we have described in detail for which disruption and altered expression of the locus would be expected to have significant consequences for neural function. Flies harboring *hobo* in *Sh* and *flea* in *cac* might show altered voltage-gated currents, whereas those with *roo* in *AstA-R1* will respond differently to the modulatory Allatostatin A neuropeptide (Smith et al. 1996; Larsen et al. 2001). We also described insertions of *412* in *teq* and *opus* in *Bx*, two genes which have been implicated in long-term memory formation (Didelot et al. 2006; Hirano et al. 2016). The *412* insertion in *teq* is particularly interesting in light of several behavioral studies that have used a mutant fly strain in which *teq* function is apparently impaired by a piggyBac transposon in the 3′ UTR (Thibault et al. 2004; Didelot et al. 2006). It seems likely that a *412* in the coding region will have at least as disruptive an effect on *teq* function as a 3′ UTR insertion.

We also discovered many cases in which a single intronic transposon introduced several cryptic splice sites, and thereby increased the transcript repertoire of the host gene. For example, the antisense *roo* inside the innate-immunity gene *mtd* resulted in many new predicted protein isoforms. This *roo* insertion could increase allele diversity and enable the innate immune system to broaden its effectiveness against a wider range of pathogens.

RNA-seq data from other fly strains suggests that more than half of the chimeric transposon transcripts identified in αβCherry flies are unique to this strain. This finding alone shows the incredible heterogeneity of transposons between strains. In addition, our prior genome sequencing revealed large differences between individual αβCherry flies (Treiber and Waddell 2017). It seems likely that polymorphic transposons and differential distribution across the genome could contribute toward heterogeneity of neural function, and neurological pathology, between individual animals.

## Methods

### Fly strains

All experiments used αβCherry flies, which were generated by crossing MB008b females (Aso et al. 2014) with w^1118^; +; UAS-mCherry males. Flies were raised on standard molasses food at 25°C, 40%–50% humidity, and 12 h:12 h light-dark cycles.

### Bulk mRNA sequencing

For RNA extraction, groups of about 50 flies were frozen in liquid nitrogen and vortexed for 6 × 30 sec to separate body segments. Heads were isolated using a sieve. To avoid gDNA contamination, mRNA was purified with a combination of protocols. Samples were first processed with a column-based kit (RNeasy Mini kit, Qiagen), including on-column DNase I digestion. Next, mRNA was extracted from total RNA using oligo(dT) magnetic beads (NEB), and mRNA was purified again using RNA columns. Finally, sequencing libraries were generated using oligo(dT) magnetic beads from a strand-specific mRNA library preparation kit (TruSeq, Illumina), with 17 cycles of PCR amplification. Fragmentation was optimized to obtain ∼350-nt-long fragments. Whole-genome sequencing was performed on a HiSeq 2500, with 250-nt paired-end reads.

### Single-cell read alignments

The *Drosophila melanogaster* reference genome release 6.25 was used for all sequence alignments (Hoskins et al. 2015). Transposon reference sequences were from Repbase (Jurka 2000; Kaminker et al. 2002). Repetitive sequences in the *Drosophila* reference genome were masked using RepeatMasker (Smit et al. 2015), and a single consensus sequence copy of each transposon was added to the reference genome. Consequently, each transposon was treated as a separate “chromosome” by the downstream analysis software. Single-cell sequencing data was processed with the Drop-seq pipeline, as described (Macosko et al. 2015; Croset et al. 2018), and Digital Gene Expression (DGE) matrices were processed using Seurat in R (R Core Team 2017; Butler et al. 2018). A detailed protocol is provided in the Supplemental Methods. The modified reference genome and refFlat file are provided as Supplemental Files 1 and 2. Mapping efficiency was assessed by comparing the number of reads mapped to consensus transposon sequences with fractional read counts estimated by RepEnrich2. Consensus reads were quantified using SAMtools idxstats on the sorted and indexed output BAM files following STAR alignment in the scTE-seq pipeline. Fractional read counts were computed using standard RepEnrich2 parameters and the most recent transposon insertion library downloaded from RepeatMasker (db20140131) for each of the eight biological replicates. Least-square linear regression was computed using GraphPad Prism (version 8) with default parameters.

### Coexpression analysis

Expression levels of every annotated gene and transposon (i.e., feature) were binarized (expression ON/OFF) in the scRNA-seq data using a dynamic threshold for UMI counts. The threshold was chosen to separate the lower third of UMI counts (OFF) from the rest (ON). Next, the coexpression disequilibrium (CD) was calculated for each transposon–gene pair as described in the main text and Figure 2A, resulting in a CD-matrix. Normalized CD values of each transposon with every feature were ranked in each replicate. For coexpression analysis, the mean ranks across all eight replicates of all features were first calculated. Next, a one-sample *t*-test was conducted with each CD value and with the expected value µ set to the mean ranks. *P*-values were corrected for multiple comparisons using Benjamini–Hochberg correction. This process was repeated with a set of 10 randomly assigned features for each transposon. Finally, a χ^2^ test was performed with the number of correlated features between each transposon and a randomly assigned feature as the expected value. Statistical analyses were performed in R.

### Mapping transposon insertions (gDNA and mRNA)

Germline transposon insertions were mapped with single-nucleotide resolution using previously published gDNA data from αβCherry flies (Treiber and Waddell 2017). Chimeric transcripts were detected by analyzing bulk mRNA data generated for this study. A new, purpose-built, multifunctional sequence analysis pipeline called TEchim was developed for both these tasks. TEchim has six key functions: (1) generation of support files, including a masked reference genome and endogenous intron–exon junctions (input files: reference genome, list of genes, list of transposon sequences); (2) alignment of unstranded genomic DNA sequence data of multiple sequencing lanes and multiple biological replicates, detection of chimeric sequence fragments with single-nucleotide resolution, the sequencing coverage around insertion sites, and the generation of summary output tables; (3) alignment of stranded cDNA data, detection of chimeric fragments, and quantification of reads; (4) generation of matching immobile genetic elements (IGEs, see main text) and analysis of these IGEs (these data are then used to determine sample-specific detection thresholds); (5) quantification of LTR-gene and LTR-transposon reads (Fig. 6B); and (6) quantification of locus-specific breakpoint-spanning reads. For key function 1, the reference genome was first masked using RepeatMasker (Smit et al. 2015) [parameters: -no_is -s] using the same library of transposon consensus sequences as for mapping the scRNA-seq data (see above). In addition, several files were created that contain information about gene features and that were required for subsequent TEchim analysis steps. For key functions 2 and 3, paired-end sequencing reads were first merged using FLASH (Magoč and Salzberg 2011) [parameters: -x 0.15 (maximum allowed ratio between the number of mismatched base pairs and the overlap length) -M 170 (maximum overlap)]. Next, in silico paired-end reads were generated from contiguous sequences. For cDNA input, the strandedness was preserved throughout the analysis. In silico reads were aligned using the STAR aligner (Dobin et al. 2013) and the masked genome (described above) [parameters: ‐‐chimSegmentMin 20 ‐‐chimOutType WithinBAM ‐‐outSAMtype BAM SortedByCoordinate]. For those in silico read pairs in which one read mapped onto a transposon sequence and their mate read mapped to a genomic locus in the masked reference genome, long-read contigs were taken and aligned to (1) the masked reference genome and (2) to consensus transposon sequences using BLAST (Altschul et al. 1990). Reads for which BLAST successfully identified alignments for both the gene and transposon breakpoint were further processed. For those cases in which only the genomic locus could be mapped, the transposon breakpoint was computed from the STAR alignment and the size of the fragment. Pooled results were filtered to ensure that each read was only counted once. These steps were repeated for each sample and sequencing lane separately and individual results were combined by merging breakpoint-spanning reads based on the genomic locus with BEDTools (Quinlan and Hall 2010), with a window of 20 nt, and preserving single-nucleotide breakpoint information on the gene- and transposon sequence. For cDNA data, transposon-to-gene and gene-to-transposon reads and for gDNA data, upstream and downstream reads were recorded separately. Pooled hits were intersected with annotated genes, gene features (5′ and 3′ UTRs, exons, introns), and splice sites. Finally, for cDNA data, gene and transposon expression levels are added to each breakpoint using SAMtools (Li et al. 2009). Key functions 4–6 are described in the Supplemental Methods. All step-by-step code and a more detailed manual are available on GitHub (https://github.com/charliefornia/TEchim). FlyBase was used for candidate-based gene searches (Thurmond et al. 2019).

### Data from previously published studies

Raw single-cell sequencing reads from Croset et al. (2018) (PRJNA428955), Hemphill et al. (2018) (PRJNA412381), and Mackay et al. (2012) (PRJNA280097) were obtained from the NCBI Short Read Archive (SRA; https://www.ncbi.nlm.nih.gov/sra). Genomic DNA data from Treiber and Waddell (2017) was obtained from the Dryad Digital Repository (https://doi.org/10.5061/dryad.fd930).

## Data access

All processed data are presented in Supplemental Tables S1–S10. FASTQ files and wiggle tracks of the bulk RNA sequencing data have been submitted to the NCBI BioProject Database (https://www.ncbi.nlm.nih.gov/bioproject/) under accession number PRJNA588978. Scripts are provided as Supplemental Code and can also be accessed via GitHub (https://github.com/charliefornia/TEchim and https://github.com/charliefornia/scHardyWeinberg).

### Competing interest statement

The authors declare no competing interests.

## Supplementary Material

Supplemental Material
