# Supplementary material for: Transposon expression in the Drosophila brain is driven by neighboring genes and diversifies the neural transcriptome
Source: Genome Res. 2020 Nov;30(11):1559–69. doi: 10.1101/gr.259200.119 (PMC7605248; doi:10.1101/gr.259200.119)
Supplement: Supplemental Material [file supp_30_11_1559__index.html]

Transposon expression in the Drosophila brain is driven by neighboring genes and diversifies the neural transcriptome — Transposon expression in the Drosophila brain is driven by neighboring genes and diversifies the neural transcriptome — Supplemental Material 

# Transposon expression in the *Drosophila* brain is driven by neighboring genes and diversifies the neural transcriptome

## Supplemental Material

- Supplemental\_Code.zip
- Supplemental\_Material\_Document.pdf
- Supplemental\_Tables\_and\_Files.zip
