# Supplementary material for: Transposon expression in the Drosophila brain is driven by neighboring genes and diversifies the neural transcriptome
Source: Genome Res. 2020 Nov;30(11):1559–69. doi: 10.1101/gr.259200.119 (PMC7605248; doi:10.1101/gr.259200.119)
Supplement: Supplemental Material [file supp_gr.259200.119_Supplemental_Material_Document.pdf]

Supplemental Material

Transposon expression in the *Drosophila* brain is driven by neighboring genes and diversifies the neural transcriptome

Christoph D. Treiber* & Scott Waddell*

Centre for Neural Circuits and Behaviour, University of Oxford, Tinsley Building, Mansfield Road, Oxford OX1 3SR, UK

*Correspondence.

Email: christoph.d.treiber@gmail.com, scott.waddell@cncb.ox.ac.uk

Table of contents:

pages 2-4: Supplemental Methods

pages 5-14: Supplemental Figures

pages 15-25: Supplemental Tables

page 26: legends for Supplemental Codes

Supplemental Methods

Processing single-cell sequencing reads

Cellular barcodes and unique molecular identifiers (UMIs) were tagged as BAM flags to reads for each sample, reads with low cellular barcode or UMI read quality score (Q10, equivalent to an error rate of 1 in 10, or lower) were removed and poly(A) tails (at least 6 contiguous As) were trimmed. Tagged reads were subsequently aligned to the masked reference genome, including the transposon consensus sequences, using STAR aligner with default settings (Dobin et al., 2013). Genes were assigned to reads using a gene reference file with annotated *Drosophila melanogaster* genes (genome release 6.25), and in addition both the sense- and antisense orientation of reference transposon sequences. Finally, Digital Gene Expression (DGE) matrices of 10,000 cells per sample were generated. The scripts used can be downloaded from GitHub (https://github.com/charliefornia /scFlyTools).

Single-cell data analysis

DGE's were first filtered (≥ 800 UMIs/cell, ≥ 400 & < 30,000 features/cell) and 8 replicates were merged. Gene and transposon expressions levels were processed separately. Read numbers were normalized, variable features were determined, and the data was scaled, using default parameters. Next, the first 80 principal components were calculated, and both a t-SNE and UMAP dimensional reduction was performed. A shared nearest neighbor graph was constructed (with 80 dimensions of reduction and default parameters) and the modularity function was optimized to determine clusters (with a resolution of 3.5). Scripts used are provided as Supplemental File 3. Marker genes were taken from Croset et al., (2018) and used to assign clusters to known cell types.

Splice acceptor (SA) and donor (SD) motif analysis

SA and SD motifs in the *Drosophila melanogaster* reference genome were generated by randomly selecting 500 known SA and SD sites from exons and screening for motifs in these sequences using the online tool MEME, which is part of the MEME suite (version 5.1.0), with default parameters (Bailey & Elkan, 1994) (Supplemental Figure S4). These motifs were then searched in sequence sections across transposon-gene breakpoints using FIMO, which is also part of the MEME suite (Grant, Bailey, & Noble, 2011).

TEchim

For key function 4 of TEchim, 10 sets of randomly chosen exons with matching expression levels in each sequencing sample were first determined. Next, function 1 (masking of reference genome) and function 2/3 (identification and quantification of breakpoint-spanning reads) was performed by replacing transposon- with IGE sequences. For key function 5, read pairs where one mate mapped onto the LTR of each tested transposon were first extracted from each sample and sequencing lane. Next, these reads were split into those with a mate mapping to a genomic locus, and those where the mate mapped onto the “core” transposon sequence (i.e. the section that is not part of the long terminal repeat). For key function 6, the total number of reads per sample and lane where one mate mapped to a genomic locus and the other one to each transposon was first extracted, using SAMtools, and in addition, the number of reads per transposon nucleotide was measured using the BEDTools function genomecov [parameters: -d]. Sequencing coverage around breakpoints was computed from TEchim output files by quantifying the reads 20 nucleotides up- and downstream of all predicted gene breakpoints using the BEDTools multicov function.

References for Supplemental Methods

Bailey, T. L., & Elkan, C. (1994). Fitting a mixture model by expectation maximization to discover motifs in biopolymers. *Proceedings. International Conference on Intelligent Systems for Molecular Biology*, *2*, 28–36. Retrieved from http://www.ncbi.nlm.nih.gov/pubmed/7584402

Croset, V., Treiber, C. D., & Waddell, S. (2018). Cellular diversity in the Drosophila midbrain revealed by single-cell transcriptomics. *ELife*, *7*(APRIL2018), 1–31. https://doi.org/10.7554/eLife.34550

Dobin, A., Davis, C. A., Schlesinger, F., Drenkow, J., Zaleski, C., Jha, S., … Gingeras, T. R. (2013). STAR: Ultrafast universal RNA-seq aligner. *Bioinformatics*, *29*(1), 15–21. https://doi.org/10.1093/bioinformatics/bts635

Grant, C. E., Bailey, T. L., & Noble, W. S. (2011). FIMO: Scanning for occurrences of a given motif. *Bioinformatics*, *27*(7), 1017–1018. https://doi.org/10.1093/bioinformatics/btr064

Supplemental Figure S1. Sense strand transposon transcripts are twice as abundant as antisense strand transcripts in the *Drosophila* midbrain.

Graph showing mean normalized expression levels across the entire midbrain of all sense and antisense transposon sequences. Each data point represents one transposon sub-family. Error bars represent the SEM.

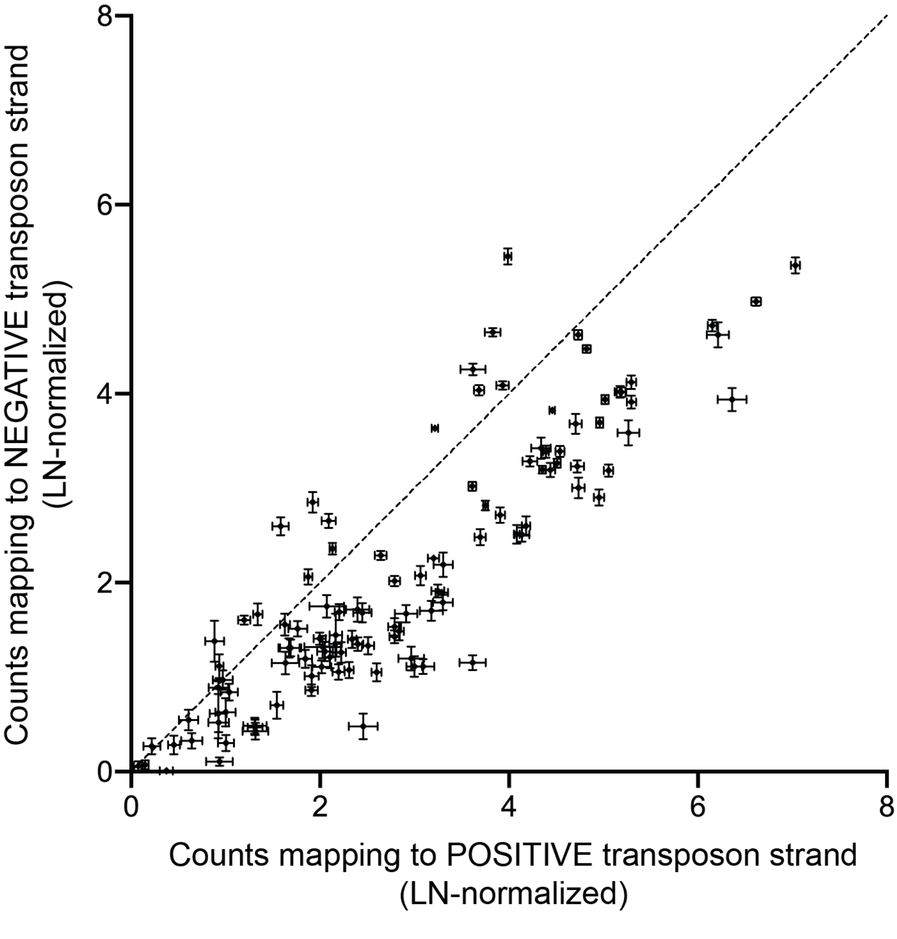

Supplemental Figure S2. Estimated expression levels of transposon sub-families in DropSeq data using STAR aligner and RepEnrich

Graph showing the mean binary logarithm counts-per-million (Log_2CPM) reads uniquely mapping to transposon consensus sequence using STAR aligner (x-axis) and the mean Log_2CPM of fractional counts assigned to each transposon sub-family by RepEnrich, using individual transposon sequences for each mapped genomic locus in the *Drosophila* reference genome (release 6.25) (y-axis). Black lines represent the least-squares linear regression and the 95% confidence intervals. A red, dashed diagonal line with slope=1 is shown for reference. Error bars represent SEM.

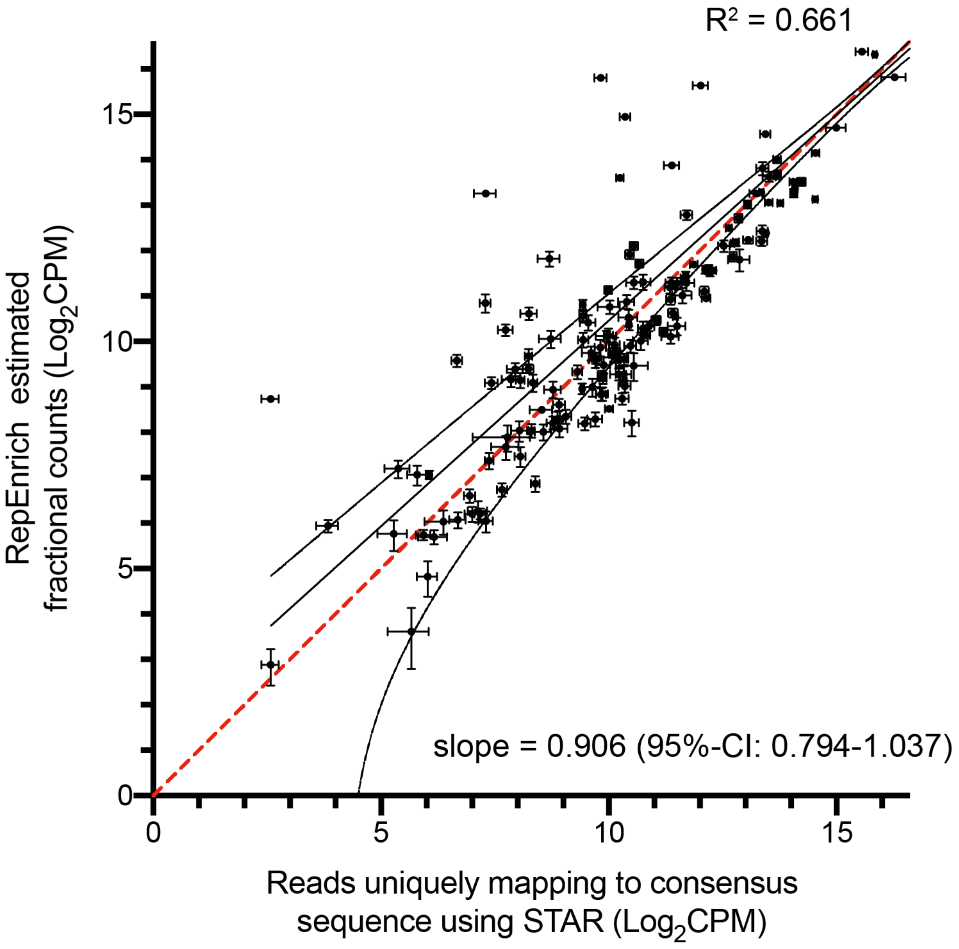

Supplemental Figure S3. Transposon expression patterns are stereotyped across biological replicates.

tSNE based on transposon expression levels showing all 8 biological replicates. Each replicate contributes cells to each cluster.

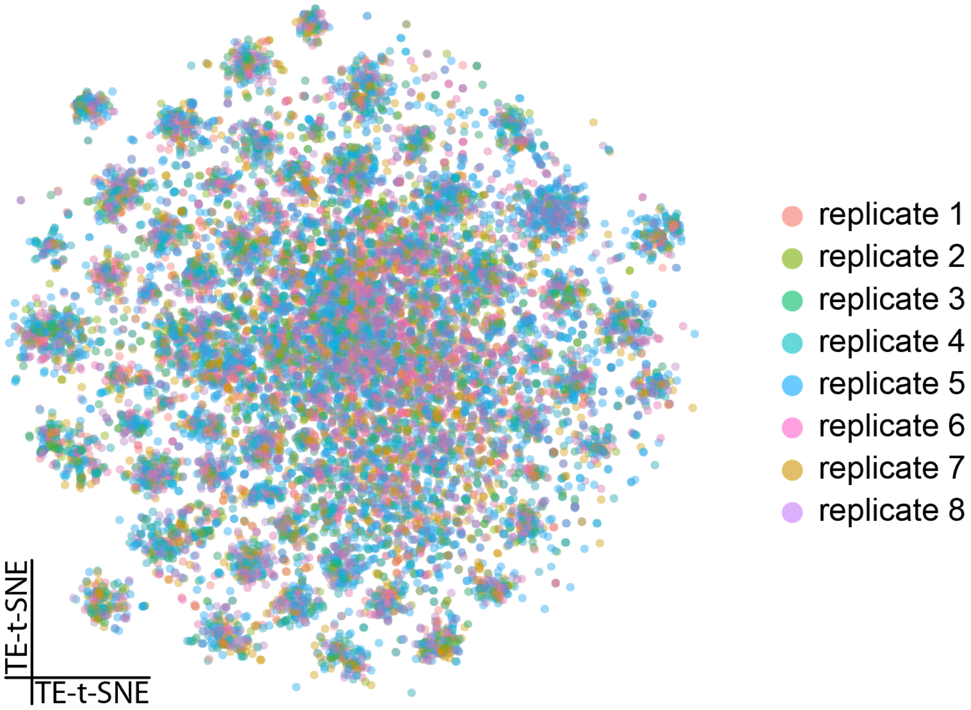

Supplemental Figure S4. Effect of different thresholds on FDR (blue) and number of detected transposon insertions (pink).

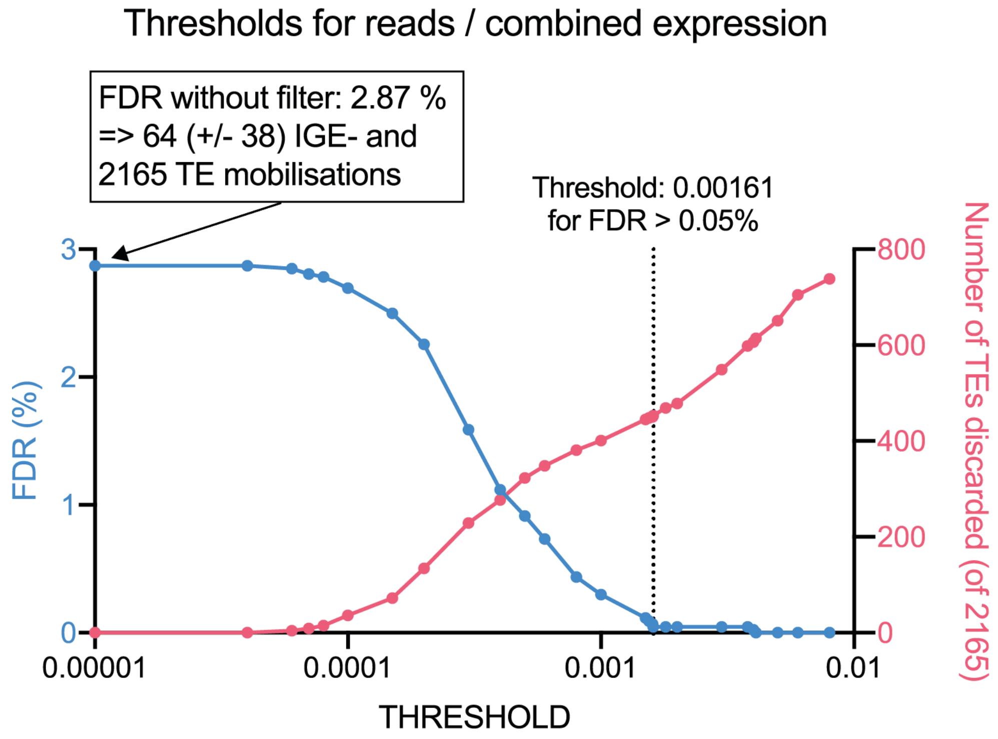

Supplemental Figure S5. Splice acceptor and donor motifs.

A Splice acceptor motif, taken from 500 randomly chosen exon-intron junctions of *Drosophila* genes. **B** Splice donor motif.

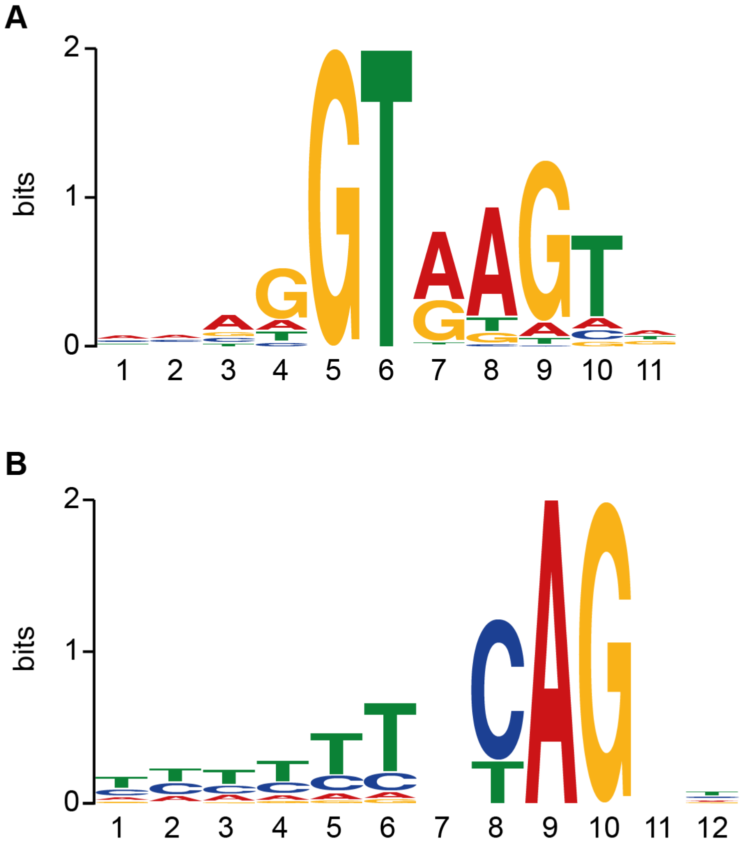

Supplemental Figure S6. Sense *blood* insertion in *Down syndrome cell adhesion molecule* (*Dscam2*).

Schematics of *Dscam2* mRNAs produced from locus containing *blood*. Top shows the nascent transcript spliced around the intronic full-length sense *blood* insertion. Bottom illustrates a new mRNA splice isoform, which reads through in frame from the ORF2 sequence of *blood* into exon 2 of *Dscam2*. The breakpoint in *blood* is a consensus SD motif. *Dscam2* also harbors a *Doc* insertion in its 5´-UTR (see Figure 5C), which is only indicated with a red dashed box for easier readability.

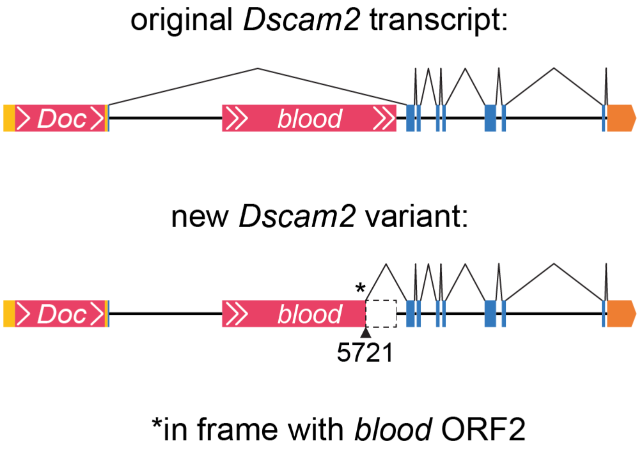

Supplemental Figure S7. Sense *412* insertion in *Tequila* (*teq*).

Schematics of *teq* mRNAs produced from locus containing full-length sense orientation *412* insertion. Top, original transcripts of *teq* splicing around *412*. Bottom, and new *teq* splice isoforms that include *412* sequence. 31.7% of *teq* transcripts contain *412* sequence. In addition, 11.3% of *412* containing mRNAs skip exon 5 of *teq*. In 0.3% of cases exons 2-5 are skipped.

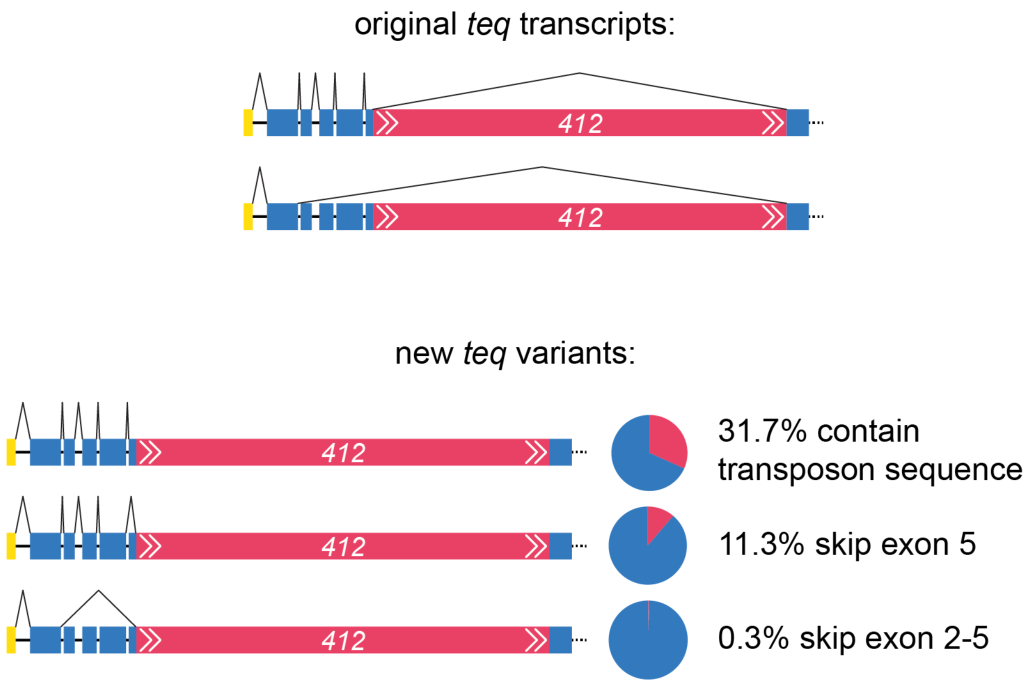

Supplemental Figure S8. Antisense *hobo* insertion in *CG31705*.

Schematic of *CG31705* mRNAs produced from locus containing exonic antisense *hobo* insertion. Transcripts containing unspliced *hobo* and two additional new splice isoforms that are generated by alternative splicing into *hobo* are shown.

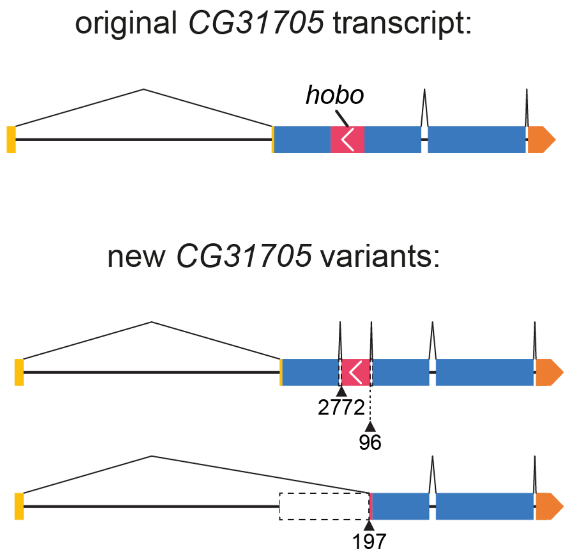

Supplemental Figure S9. Sense *flea* insertion in *cacophony* (*cac*).

Schematic *cac* transcripts produced from locus containing a full-length intronic sense *flea* insertion (the orientation is 5´ (left) to 3´ (right)). A regular *cac* transcript is produced by splicing around the *flea* insertion and a new truncated *cac* isoform results from splicing into *flea*.

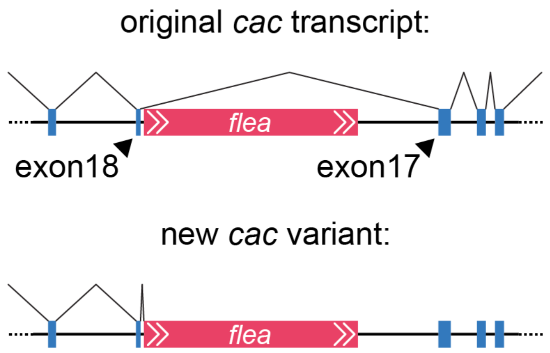

Supplemental Figure S10. Sense *opus* insertion in *Beadex* (*Bx*).

Schematic showing mRNAs produced from locus containing sense intronic *opus* insertion. Original transcript of *Bx* is generated by splicing around *opus*. New splice isoforms contain fragments of *opus*.

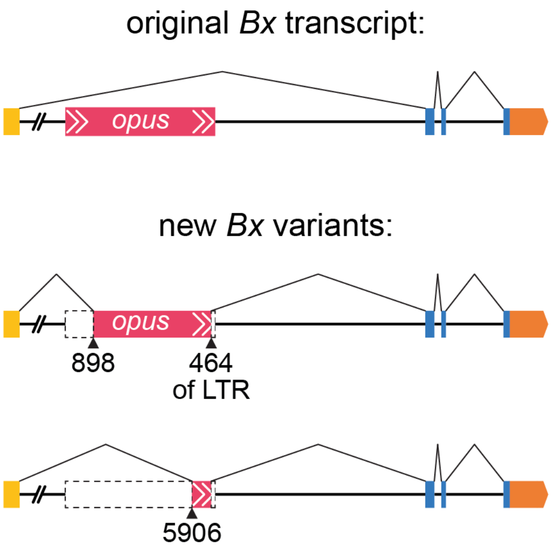

Supplemental Table S1 (EXTRACT): Transposon expression levels in an average cell per biological replicate. All transposons contributing to >1% of overall transposon expression are shown. See Supplemental_Table_S1.csv for the full list.

TE	Transposon expression level (average across all cells)								Combined average	Relative expression
	Repl. 1	Repl. 2	Repl. 3	Repl. 4	Repl. 5	Repl. 6	Repl. 7	Repl. 8		
copia	3.56	2.60	5.43	6.22	4.99	4.30	5.06	4.08	4.53	11.44
roo	2.63	1.44	2.88	3.55	3.49	2.72	3.88	3.47	3.01	7.59
Tabor	2.66	1.81	1.52	2.22	1.04	3.16	3.68	3.30	2.42	6.12
opus	1.23	1.52	1.71	2.41	1.12	1.86	3.74	3.24	2.10	5.31
Doc	1.37	1.00	1.94	2.45	2.29	1.70	2.68	1.79	1.90	4.80
NEG_hopper	0.85	0.53	0.70	1.07	0.64	1.19	1.40	1.20	0.95	2.39
NEG_copia	0.60	0.71	1.24	1.17	0.87	0.67	0.83	0.70	0.85	2.14
F-element	0.60	0.41	0.87	1.04	1.01	0.69	0.96	0.85	0.80	2.03
mdg1	0.85	0.65	0.52	0.80	0.44	0.86	1.23	0.91	0.78	1.97
hobo	0.68	0.68	0.74	0.93	0.92	0.64	0.92	0.72	0.78	1.96
I-element	0.51	0.36	0.86	0.85	0.74	0.74	0.89	0.80	0.72	1.82
mdg3	0.65	0.64	0.59	0.82	0.65	0.58	0.91	0.67	0.69	1.74
invader2	0.42	0.34	0.59	0.89	0.74	0.51	0.87	0.71	0.63	1.60
412	0.57	0.37	0.52	0.86	0.55	0.57	0.74	0.60	0.60	1.51
HMS-Beagle	0.33	0.35	0.51	0.77	0.57	0.53	0.80	0.70	0.57	1.45
blood	0.47	0.35	0.52	0.78	0.65	0.47	0.79	0.52	0.57	1.44
NEG_roo	0.51	0.44	0.62	0.76	0.57	0.50	0.64	0.49	0.57	1.43
T-element	0.36	0.26	0.48	0.61	0.58	0.47	0.67	0.57	0.50	1.26
flea	0.28	0.29	0.45	0.77	0.55	0.35	0.54	0.47	0.46	1.17
Doc3-element	0.35	0.21	0.47	0.64	0.61	0.33	0.60	0.46	0.46	1.16
HeT-A	0.33	0.25	0.50	0.51	0.52	0.46	0.59	0.45	0.45	1.14
NEG_Doc	0.42	0.33	0.51	0.58	0.49	0.38	0.49	0.33	0.44	1.11
jockey	0.33	0.27	0.45	0.47	0.63	0.33	0.50	0.54	0.44	1.11
NEG_opus	0.18	0.46	0.41	0.58	0.26	0.30	0.70	0.50	0.42	1.07
NEG_Cr1a	0.39	0.32	0.43	0.54	0.40	0.32	0.48	0.39	0.41	1.03
NEG_HeT-A	0.25	0.31	0.36	0.48	0.39	0.46	0.53	0.43	0.40	1.01

Supplemental Table S2 (EXTRACT): Germline transposon insertions detected by TEchim in αβCherry flies. Hits are separated into those spanning the junction up- and downstream of the insertion site. TE strand refers to the orientation of the transposon in relation to the reference genome, not the neighboring gene. Supplemental_Table_S2.csv also includes sequencing coverage of each tested sample. Breakpoints with the ten highest breakpoint-spanning read counts are shown here. The full list comprises 16,197 hits.

Chr	Breakpoint	Transposon	fragment	TE strand	Sam-ples	Reads total	Overlapping features	Indi-viduals	Mean coverage
X	22099746	*baggins*	upstream	negative	19	4363	.(.)	10	15
3R	3472379	*Stalker4 LTR*	upstream	negative	19	3302	intron|FBgn0010247	10	173
X	21979374	*Quasimodo*	upstream	negative	19	3262	.(.)	10	83
X	23239718	*R1A1-element*	downstream	positive	19	2860	pseudogene|FBgn0085771	10	1264
2L	23022162	*Quasimodo LTR*	upstream	positive	19	2732	intron|FBgn0250907<br>intron|FBgn0250907<br>intron|FBgn0250907	10	191
X	23271158	*R1A1-element*	upstream	negative	19	2624	pseudogene|FBgn0267520	10	1280
X	22275317	*baggins*	upstream	negative	19	2556	.(.)	10	225
X	23421137	*G-element*	downstream	negative	18	2367	.(.)	10	2123
X	22276843	*baggins*	downstream	negative	19	2333	.(.)	10	299
Y	1416648	*GATE*	downstream	positive	19	2305	.(.)	10	74
X	21822292	*Quasimodo LTR*	upstream	negative	19	2238	.(.)	10	254
Y	1416803	*GATE*	upstream	positive	19	2222	.(.)	10	70
X	22275723	*baggins*	downstream	negative	19	2135	.(.)	10	257
X	23217090	*R2-element*	upstream	positive	19	2118	pseudogene|FBgn0267519	10	1559
X	23217141	*R1A1-element*	upstream	positive	20	2020	.(.)	10	1151
2R	2009037	*invader5 LTR*	downstream	positive	18	1921	intron|FBgn0263780<br>intron|FBgn0263780	10	109
3L	23133913	*Quasimodo*	downstream	negative	19	1857	.(.)	10	87
X	22269981	*baggins*	upstream	negative	18	1853	.(.)	10	28
2L	23483977	*Circe*	downstream	positive	19	1629	.(.)	10	29

Supplemental Table S3 (EXTRACT): List of transposons found inserted in a gene in the gDNA of at least 5 tested individuals, and the according gene. P-values reflect the probability that two independent features would have a Coexpression Disequilibrium value across all replicates as high as each individual transposon-gene pair. Only the feature pair with the lowest p-value for every transposon that contributes at least 1% of the overall transposon expression is shown. Supplemental_Table_S3.csv contains the full list of pairs, and in addition p-values of 10 randomly assigned genes for each transposon insertion.

Transposon	Relative expression	Neighboring gene	P-value	Corrected p-value	Below threshold	10 x random genes
copia	11.44242262	*Ten-a*	0.0000027	0.0001957	yes	…
roo	7.594725396	*dnc*	0.0000039	0.0001957	yes	…
Tabor	6.121253992	*Bsg*	0.0000190	0.0002555	yes	…
Doc	4.802139806	*Gfrl*	0.0000017	0.0001957	yes	…
opus	5.311921907	*mub*	0.0000285	0.0002914	yes	…
NEG_hopper	2.394921941	*CR44999*	0.0000045	0.0001957	yes	…
hobo	1.962582927	*Sh*	0.0000040	0.0001957	yes	…
mdg1	1.974034086	*dpr8*	0.0000075	0.0002063	yes	…
F-element	2.026828928	*dpr8*	0.0000098	0.0002063	yes	…
mdg3	1.743184084	*SelR*	0.0000275	0.0002902	yes	…
NEG_copia	2.142216363	*Shawl*	0.0000072	0.0002063	yes	…
I-element	1.817248786	*Snap25*	0.0000153	0.0002367	yes	…
blood	1.43625691	*bru3*	0.0000339	0.0003256	yes	…
412	1.511605763	*Sh*	0.0000064	0.0002063	yes	…
HMS-Beagle	1.445076291	*Ssdp*	0.0000111	0.0002093	yes	…
invader2	1.602389348	*CG17684*	0.0000044	0.0001957	yes	…
NEG_roo	1.432608183	*Sh*	0.0000040	0.0001957	yes	…
jockey	1.112244173	*bru3*	0.0000033	0.0001957	yes	…
NEG_Doc	1.113936251	*CG17514*	0.0000023	0.0001957	yes	…
Doc3-element	1.159078004	*CG17684*	0.0000118	0.0002134	yes	…
flea	1.165831288	*cac*	0.0000061	0.0002063	yes	…
NEG_Cr1a	1.031149519	*Myo81F*	0.0000044	0.0001957	yes	…

Supplemental Table S4 (EXTRACT): List of transposons, sorted by expression level in scRNA-seq data, with the total number of correlated neighboring genes, and the mean number of randomly assigned genes that were also correlated. P-values were calculated using a chi-squared test and rates of randomly correlated genes were used as the expected values for each transposon. See Supplemental_Table_S4.csv for full list of transposons.

Transposon	Neighboring gene		10 random genes (mean)		P-value (chi-squared test)	Transposon expression levels
	not correlated	correlated	not correlated	correlated		
copia	6	8	12.8	1.2	8.47E-11	9.599
roo	32	43	63.6	11.4	2.89E-24	6.604
Tabor	1	3	3.7	0.3	2.97E-07	6.020
Doc	23	19	36.5	5.5	6.62E-10	4.651
opus	9	6	13.9	1.1	1.21E-06	3.514
NEG_hopper	8	15	19.9	3.1	3.70E-13	2.365
hobo	16	20	32.3	3.7	3.68E-19	1.999
mdg1	10	4	12.5	1.5	3.08E-02	1.904
F-element	11	16	23.8	3.2	2.51E-14	1.743
mdg3	2	6	7	1	9.03E-08	1.685
NEG_copia	9	10	17.7	1.3	2.67E-15	1.580
I-element	6	5	9.9	1.1	8.87E-05	1.560
blood	10	7	15.8	1.2	3.97E-08	1.551
412	8	7	13.5	1.5	2.21E-06	1.505
HMS-Beagle	3	1	3.4	0.6	5.75E-01	1.367
invader2	2	7	8.4	0.6	1.21E-17	1.330
NEG_roo	43	48	81.8	9.2	1.74E-41	1.145
jockey	14	20	31.2	2.8	7.34E-27	1.062
NEG_Doc	27	26	46.4	6.6	6.99E-16	1.052
Doc3-element	8	5	11.7	1.3	6.25E-04	0.979
flea	3	4	6.7	0.3	5.03E-12	0.962
NEG_Cr1a	21	23	40.1	3.9	4.02E-24	0.924

Supplemental Table S5 (EXTRACT): Combined TEchim output of gDNA and mRNA data. Hits on Chromosome 3R between 5274800 and 5292640. "Fragment" shows strand-specific data for mRNA, and indicates junction side in relation to transposon in gDNA data. "TE orientation" refers to the actual transcript for mRNA data, and the genomic strand for gDNA data. "Above threshold" refers to the threshold in Figure 3A. "TE ratio" is the percentage of reads indicative of a chimeric transcript vs. reads that span two chromosomal exons without transposon sequence (used e.g. for graphs in Figure 5). Supplemental_Table_S5.csv contains all hits, and additional information.

Data source	Gene	Chr	Str-and	Break-point	Trans-poson	Frag-ment	TE orientation	Sam-ples	Reads total	Splice site	TE break-points	Above threshold	TE present (mean)	TE absent (mean)	TE ratio
mRNA	*mtd*	3R	-	5274800	*roo*	TE-GENE	minus	1	1	.	641(1)				
mRNA	*mtd*	3R	-	5274895	*roo*	TE-GENE	minus	2	2	5274894	5107-5192(1) 5190(1)	no	2.17	528	0.41
mRNA	*mtd*	3R	-	5275043	*roo*	TE-GENE	minus	1	1	.	5192(1)				
mRNA	*mtd*	3R	-	5275088	*roo*	TE-GENE	minus	6	34	5275087	2781(4) 5190(27) 5191(1) 639(2)	yes	5	445.67	1.11
mRNA	*mtd*	3R	-	5283984	*roo*	TE-GENE	minus	1	1	.	5191(1)				
gDNA		3R	.	5284269	*roo LTR*	down-stream	positive	14	46		428(46)				
mRNA	*mtd*	3R	-	5284269	*roo LTR*	GENE-TE	minus	4	21	.	1(2) 428(19)	yes	4	202.5	1.94
mRNA	*mtd*	3R	-	5284273	*roo LTR*	TE-GENE	minus	6	42	.	1(42)	yes	7.67	79.83	8.76
gDNA		3R	.	5284273	*roo LTR*	up-stream	positive	11	34		1(33) 13-1(1)				
mRNA	*mtd*	3R	-	5286581	*roo*	GENE-TE	minus	2	2	.	2811(1) 2853(1)	no	1.17	345.17	0.34
mRNA	*mtd*	3R	-	5286630	*roo*	GENE-TE	minus	2	2	.	2811(1) 2973(1)	no	1.33	359.67	0.37
mRNA	*mtd*	3R	-	5286902	*roo*	GENE-TE	minus	2	5	5286902	2095(1) 5206(1) 5458(1) 5461(2)	yes	1.17	315.33	0.37
mRNA	*mtd*	3R	-	5291990	*roo*	GENE-TE	minus	1	1	5291990	5462(1)				
mRNA	*mtd*	3R	-	5292640	*roo*	GENE-TE	minus	6	15	5292640	5462(15)	yes	3.5	255.67	1.35

Supplemental Table S6 (EXTRACT): Combined expression level for each transposon-gene pair and IGE-gene pair was calculated and plotted against the total number of reads supporting a chimeric transcript. This table only shows the top-ranked feature pair for every set. See Supplemental_Table_S6.csv for the entire list and Figure 3A for a graphical representation of the data.

Combined expression	Total number of reads supporting chimeric transcript										
	Transposons	IGE run1	IGE run2	IGE run3	IGE run4	IGE run5	IGE run6	IGE run7	IGE run8	IGE run9	IGE run10
355	1511										
7584		6									
44210			14								
24800				8							
26085					5						
12002						3					
12936							15				
26085								5			
37572									10		
41110										12	
1162											4

Supplemental Table S7 (EXTRACT): Results of FIMO (MEME-suite) for all transposon sections that were detected to form chimera with intron-exon junctions. The 30 sequences with the lowest p-value are shown. See Supplemental_Table_S7.csv for full list.

Transposon	TE strand	Breakpoint	Motif	P-value	Q-value	Matched sequence
roo	-	5460	splice_accetor_site	2.63E-06	0.000209	tttcttgcagct
Doc	-	2555	splice_donor_site	3.92E-06	0.0185	acaggtgagtg
roo	-	2094	splice_accetor_site	1.09E-05	0.000753	ttctttacaggt
Juan	-	3908	splice_donor_site	1.25E-05	0.0295	taaggtaagtt
flea	+	1826	splice_accetor_site	1.27E-05	0.013	tcttttacagtt
diver	+	4466	splice_accetor_site	6.94E-05	0.0473	ctatttacagat
17.6	-	5671	splice_accetor_site	0.00011	0.00695	tctatttcagtt
blood	-	4832	splice_accetor_site	0.000122	0.00695	ttgttttcagat
Juan	-	1163	splice_donor_site	0.000128	0.0865	attggtgagtg
Doc	+	2366	splice_donor_site	0.00015	0.0575	aagggtaagca
roo	-	2783	splice_donor_site	0.000178	0.093	gaatgtgagta
copia	-	2423	splice_accetor_site	0.000204	0.0105	tatctttcagga
copia	-	2531	splice_accetor_site	0.000204	0.0105	tatctttcagga
P-element	+	442	splice_donor_site	0.000246	0.0575	cagagtaagtt
jockey	-	4332	splice_donor_site	0.000276	0.0976	gaaggtgagct
opus_LTR	+	460	splice_donor_site	0.000276	0.0575	caaggtgaggt
blood	-	4729	splice_donor_site	0.00029	0.0976	acgagtgagtt
copia	+	141	splice_accetor_site	0.000299	0.122	tccttctcagaa
Tc1-2	-	1126	splice_accetor_site	0.0003	0.0145	catctttcagaa
jockey	-	4499	splice_accetor_site	0.00036	0.0169	ctcttcgcagca
Doc	-	336	splice_donor_site	0.0004	0.103	gaaggtgaggg
invader1	+	952	splice_donor_site	0.0004	0.0739	tctggtgagtc
hobo	-	197	splice_accetor_site	0.000404	0.0184	cccttttaggc
mdg3_LTR	-	51	splice_donor_site	0.000408	0.103	ttaggtatgta
Transpac_LTR	-	22	splice_donor_site	0.000437	0.103	gagggtatgta
mdg3_LTR	+	160	splice_accetor_site	0.000456	0.143	cactctgcagag
transib1	-	321	splice_donor_site	0.0005	0.11	atcagtaagtt
Burdock	+	200	splice_donor_site	0.00053	0.0803	tattgtaagta
412	-	5689	splice_donor_site	0.000559	0.11	acaggtcagta
mdg1_LTR	-	335	splice_accetor_site	0.000693	0.0299	tacattccagat

Supplemental Table S8 (EXTRACT): Combined TEchim output from mRNA data of 4 different fly strains. Shown here are 2 representative examples of transposon-gene pairs. See Supplemental_Table_S8.csv for the full list.

Study	Gene	Chr	Str-and	Break-point	Trans-poson	Frag-ment	TE orien-tation	Sam-ples	Reads	Gene splicesite	Chimera ratio
Hempshill 2018	*CHKov1*	3R	-	25324411	*Doc*	TE-GENE	plus	1	1	25324410	
MacKay 2012	*CHKov1*	3R	-	25324411	*Doc*	TE-GENE	plus	2	18	25324410	46.2
THIS STUDY	*CHKov1*	3R	-	25324411	*Doc*	TE-GENE	plus	6	26	25324410	2.7
Croset 2017	*CHKov1*	3R	-	25328939	*Doc*	GENE-TE	plus	2	4	.	100.0
Hempshill 2018	*CHKov1*	3R	-	25328939	*Doc*	GENE-TE	plus	4	21	.	100.0
MacKay 2012	*CHKov1*	3R	-	25328939	*Doc*	GENE-TE	plus	2	100	.	83.0
THIS STUDY	*CHKov1*	3R	-	25328939	*Doc*	GENE-TE	plus	6	95	.	37.7
Croset 2017	*CHKov1*	3R	-	25329058	*Doc*	GENE-TE	plus	2	2	25329061	13.4
THIS STUDY	*CHKov1*	3R	-	25329058	*Doc*	GENE-TE	plus	6	349	25329061	24.3
Croset 2017	*Bx*	X	+	18521039	*opus*	GENE-TE	plus	2	14	18521039	12.6
Hempshill 2018	*Bx*	X	+	18521039	*opus*	GENE-TE	plus	1	1	18521039	
Croset 2017	*Bx*	X	+	18521195	*opus*	GENE-TE	plus	1	15	.	
THIS STUDY	*Bx*	X	+	18535221	*opus*	GENE-TE	plus	2	2	.	0.3
Hempshill 2018	*Bx*	X	+	18535424	*opus*	GENE-TE	plus	4	11	18535424	7.2
THIS STUDY	*Bx*	X	+	18535424	*opus*	GENE-TE	plus	5	13	18535424	4.6
Hempshill 2018	*Bx*	X	+	18550612	*opus LTR*	TE-GENE	plus	1	1	.	
THIS STUDY	*Bx*	X	+	18550612	*opus LTR*	TE-GENE	plus	4	11	.	6.4
Hempshill 2018	*Bx*	X	+	18550614	*opus LTR*	GENE-TE	plus	3	5	.	7.8
Croset 2017	*Bx*	X	+	18566694	*opus LTR*	TE-GENE	plus	1	2	18566696	
THIS STUDY	*Bx*	X	+	18566694	*opus LTR*	TE-GENE	plus	2	2	18566696	0.2
Hempshill 2018	*Bx*	X	+	18566695	*opus*	TE-GENE	plus	1	1	18566696	

Supplemental Table S9 (EXTRACT): Comparison of reads consistent with chimeric transcripts and total number of reads mapping to each transposon sub-family. The 30 transposons with the highest number of breakpoint-spanning reads are shown. Supplemental_Table_S9.csv includes the full list of transposons, and in addition values for each sample.

Transposon	Average number of reads spanning transposon-gene junction	Average number of reads per TE nucleotide
copia	281.67	85.03
Doc3-element	281.03	161.63
copia1	270.78	135.35
copia_LTR	181.77	88.54
transib1	162.14	111.02
Tirant_LTR	154.80	107.73
Tc1	129.54	106.75
Doc4-element	127.61	85.11
Transpac_LTR	120.38	84.26
transib3	115.46	110.68
transib5	108.65	87.74
Porto1	101.30	81.19
Doc	99.08	61.84
accord	98.90	13.08
Doc2-element	93.12	59.45
Rt1c	90.47	50.78
Stalker4_LTR	74.22	37.56
gtwin_LTR	73.41	37.80
Transpac	62.99	24.40
blood_LTR	62.44	13.10
gypsy	56.79	27.40
Stalker4	56.07	18.71
blood	54.25	14.16
TART-A	54.02	28.80
McClintock_LTR	52.88	18.79
gypsy_LTR	50.97	29.12
F-element	46.03	12.95
Rt1a	45.95	14.92
roo	45.11	25.11
flea_LTR	43.64	13.14

Supplemental Table S10 (EXTRACT): Number of reads spanning the up- and downstream LTRs. Mean read numbers are shown. Ratios are capped at 100%. See Supplemental_Table_S10.csv for values of individual replicates and un-capped ratios.

TE	GENE-LTR	LTR-TE	Chimeric ratio upstream	TE-LTR	LTR-GENE	Chimeric ratio downstream
accord	15.50	189.67	7.6%	133.33	26.33	>100%
accord2	0.50	0.83	37.5%	0.50	3.50	12.50%
GATE	0.50	1.50	25.0%	0.33	0.33	50.00%
3S18	11.50	22.83	33.5%	112.33	7.00	>100%
flea	24.67	48.17	33.9%	76.50	23.17	>100%
blood	84.67	112.83	42.9%	174.33	51.00	>100%
Burdock	13.67	127.67	9.7%	154.67	39.50	>100%
Chimpo	1.33	5.67	19.0%	3.50	3.83	47.73%
Chouto	0.83	0.00	>100%	2.17	7.33	22.81%
copia	179.67	1292.17	12.2%	1526.50	309.50	>100%
copia1	4.50	11.50	28.1%	5.83	2.33	>100%
Dm88	1.83	1.33	>100%	4.50	13.67	24.77%
diver	70.00	11.17	>100%	22.50	9.00	>100%
diver2	63.17	2.33	>100%	2.83	4.50	38.64%
1731	2.83	3.17	47.2%	4.33	2.67	>100%
17.6	30.33	52.67	36.5%	53.67	40.50	>100%
297	132.50	57.33	>100%	38.83	65.00	37.40%
412	270.17	265.00	>100%	250.50	132.67	>100%
frogger	0.67	2.67	20.0%	2.00	0.00	>100%
gtwin	0.67	7.00	8.7%	1.67	3.67	31.25%
gypsy	162.33	294.50	35.5%	610.00	138.50	>100%
springer	45.33	1.67	>100%	15.50	19.17	44.71%
gypsy10	2.17	0.50	>100%	0.83	0.67	>100%
gypsy11	2.33	1.00	>100%	0.67	2.83	19.05%
gypsy12	7.33	0.00	>100%	0.00	12.00	0.00%
gypsy2	25.83	0.33	>100%	0.33	10.33	3.13%
gypsy3	0.33	0.17	>100%	0.00	7.67	0.00%
gypsy4	1.50	4.17	26.5%	5.50	1.67	>100%
gypsy5	0.83	0.67	>100%	1.50	1.00	>100%
gypsy6	1.33	0.00	>100%	0.33	3.33	9.09%
gypsy7	0.33	0.17	>100%	0.17	0.00	>100%
gypsy8	0.17	0.00	>100%	0.00	1.33	0.00%
gypsy9	0.83	0.17	>100%	0.50	0.17	>100%
Idefix	29.83	0.00	>100%	64.83	36.50	>100%
invader1	7.00	6.33	>100%	10.50	29.00	26.58%
invader2	14.50	30.50	32.2%	22.17	16.00	>100%
invader3	0.83	0.50	>100%	6.33	2.33	>100%
invader4	83.17	17.83	>100%	9.17	60.33	13.19%
invader5	1.00	0.17	>100%	0.00	0.00	N/A
invader6	0.33	0.00	>100%	0.00	2.17	0.00%
Max-element	10.67	22.50	32.2%	2.00	7.50	21.05%
mdg1	14.50	211.50	6.4%	9.83	69.50	12.39%
mdg3	126.00	182.50	40.8%	288.50	309.50	48.24%
micropia	16.83	48.50	25.8%	109.50	157.33	41.04%

opus	70.83	262.00	21.3%	63.17	35.50	>100%
Quasimodo	61.67	33.00	>100%	80.67	294.50	21.50%
McClintock	10.67	6.50	>100%	14.17	253.67	5.29%
roo	588.00	230.17	>100%	254.67	540.00	32.05%
rooA	20.00	12.17	>100%	5.33	139.83	3.67%
Stalker2	53.50	28.50	>100%	140.50	75.83	>100%
Stalker4	7.33	0.83	>100%	11.33	21.83	34.17%
Tabor	18.67	28.00	40.0%	9.67	14.50	40.00%
Tirant	1.33	3.00	30.8%	6.00	1.33	>100%
Transpac	132.83	676.33	16.4%	396.83	123.67	>100%
ZAM	2.17	0.33	>100%	0.67	1.83	26.67%

Supplemental Code S1 [TEchim_buildREF.sh]: Code to generate required support files from a given reference genome, gene annotation file and transposon consensus sequences.

Supplemental Code S2 [TEchim_part1.sh]: Code for the first part of the TEchim analysis. This code snippet can be run on individual sequencing runs. Biological replicates as well as separate sequencing lanes of the same sample are automatically named accordingly for downstream analysis.

Supplemental Code S3 [TEchim_part2_mRNA]: Code to run TEchim on output from part1 if input was cDNA reads.

Supplemental Code S4 [TEchim_part2_gDNA]: Code to run TEchim on output from part1 if input was gDNA reads.

Supplemental Code S5 [TEchim_IGE]: Code to generate IGE run. This code can only be run after TEchim_part2_mRNA.sh, because it uses genome coverage in all samples for the choice of matching IGEs (see Methods for details).

Supplemental Code S6 [TEchim_ TEcoverage_vs_TEgeneREADS.sh]: Code used to generate plot in Figure 6a. The number of chimeric reads for each transposon subtype and the total read coverage normalized to transposon length is measured for each sample.

Supplemental Code S7 [TEchim_LTR-TE_vs_LTR-gene.sh]: Code used to generate plot in Figure 6b. The number of reads spanning each LTR sequence and either a neighboring gene or transposon subtype is measured for each sample.

Supplemental Code S8 [TEchim_postprocessing.sh]: Collection of code snippets that were used to generate Supplemental Data and Tables.
